# Nickel-Based Nanoparticles Synthesized by Pulsed Laser Ablation in Liquid with Multiphase Structure for Electrochemical Dopamine Sensing

**DOI:** 10.1021/acsomega.6c05305

**Published:** 2026-06-20

**Authors:** Tomas Raphael Woida, Philipi Cavalcante Ricardo, Caio Raphael Vanoni, Adriano Rogério Silva Lima, Kurosch Rezwan, Cristiane Luisa Jost, Márcio Celso Fredel

**Affiliations:** † Advanced Ceramics, 9168 University of Bremen, 28359 Bremen, Germany; ‡ Núcleo de Pesquisas em Materiais Cerâmicos e Compósitos (CERMAT), Departamento de Engenharia Mecânica, 28117 Universidade Federal de Santa Catarina, 88040-900 Florianópolis, SC, Brazil; § Ampere—Laboratório de Plataformas Eletroquímicas, Departamento de Química, Universidade Federal de Santa Catarina, 88040-900 Florianópolis, SC, Brazil

## Abstract

Nickel-based nanoparticles (NiNPs) synthesized by pulsed laser ablation in liquid (PLAL) were investigated as a green electrocatalytic phase for dopamine (DA) sensing. NiNPs were produced directly in ultrapure water and combined with Nafion, and drop-cast onto a glassy carbon electrode (GCE) to obtain a hydroxide-rich nickel-based/ionomer composite film. Transmission electron microscopy, X-ray diffraction, dynamic light scattering, and zeta-potential measurements confirmed a multiphase composition predominantly comprising Ni­(OH)_2_, with minor metallic Ni and NiO domains, and revealed that incorporation into the sulfonated polymer reverses the nanoparticle surface charge from positive to negative. This charge inversion enhances the preconcentration and electrocatalytic oxidation of protonated dopamine at the composite interface. Cyclic voltammetry (CV) and electrochemical impedance spectroscopy (EIS) indicate a quasi-reversible electron transfer governed by a mixed adsorption–diffusion mechanism. Under optimized conditions in Britton–Robinson (B–R) buffer (pH 3.0), the cathodic peak current varies linearly with dopamine concentration between 0.25 and 100 μmol L^–1^, with a detection limit of 92 nmol L^–1^. The sensor exhibits good repeatability and tolerance to common urinary interferents, and enables accurate determination of dopamine in synthetic human urine, with recoveries between 93.5 and 104.4%. These results demonstrate that PLAL-derived NiNPs provide an environmentally friendly and efficient platform for electrochemical monitoring of DA.

## Graphical abstract

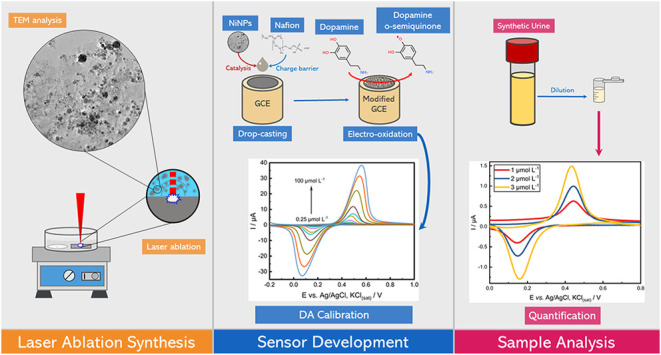

## 1 Introduction

NiNPs are emerging as versatile nanomaterials with distinctive properties, including magnetism,^1^ superparamagnetism,^2^ high catalytic efficiency,^3^ and electrical conductivity.^4^ Solution-based reduction of metal salts in aqueous or organic solvents, utilizing NiCl_2_ as the nickel source, remains a prevalent approach for synthesizing NiNPs.^5^ The conversion of Ni^2+^ to Ni^0^ is typically achieved through reducing agents such as hydrazine, sodium borohydride (NaBH_4_), or potassium borohydride (KBH_4_).^6,7^ Alternative routes include solvothermal synthesis^8^ and sonochemical methods (ultrasonic-driven reduction).^9^ Nonetheless, the synthesis of NiNPs presents challenges that require attention to enable scalable applications.

PLAL is a green and increasingly applied synthesis technique for nanoparticle production that involves focusing high-energy laser pulses onto a metal target submerged in a liquid medium. The laser rapidly heats the target, generating a plasma plume that vaporizes and ejects metal ions into the surrounding liquid, nucleating them and forming nanoparticles (NPs) with controlled size and composition.^10^ This method eliminates the need for toxic chemical reagents, significantly reducing environmental risks compared to traditional chemical reduction methods, which may produce hazardous waste and require energy-intensive purification steps.^11,12^ PLAL also avoids using organic solvents and high-temperature processes inherent to solvothermal synthesis, minimizing volatile organic compound emissions and thermal energy demands. Additionally, unlike sonochemical methods that rely on ultrasonic waves and often require surfactants, PLAL produces ″bare″ NPs with clean surfaces, enhancing their catalytic and biomedical utility by eliminating postsynthesis purification.^13^ The liquid medium in PLAL can further functionalize NPs in situ, offering tunable surface properties without additional steps.^14,15^ Therefore, PLAL’s lack of toxic byproducts, scalability, and versatility position it as a sustainable alternative for synthesizing high-purity, eco-friendly metal NPs for applications in catalysis, biomedicine, and environmental remediation.

DA is predominantly synthesized in the substantia nigra region of the brain. Additional production occurs in areas such as the ventral tegmental region and hypothalamus.^16,17^ This neurotransmitter is critically involved in regulating motor function, reward processing, emotional states, and cognitive processes, playing essential roles in both physiological and psychological functions.^18^ Its synthesis begins with the amino acid tyrosine, which is converted to levodopa and subsequently to DA via enzymatic reactions.^19^ Dysregulation of DA levels is implicated in neurodegenerative and psychiatric disorders, including Parkinson’s disease, schizophrenia, and addiction.^17,20,21^ The accurate determination of DA concentrations is critical for diagnosing these conditions, optimizing therapeutic interventions, and advancing neurochemical research.

DA can be quantified using various analytical techniques, each with its advantages and limitations. High-performance liquid chromatography (HPLC) coupled with electrochemical detection, a diode array detector, or a mass spectrometer remains a standard due to its high sensitivity and specificity, although it requires complex sample preparation and costly instrumentation.^22–24^ Electrochemical sensors, such as carbon-based or nanoparticle-modified electrodes, offer rapid, real-time detection with low detection limits and are ideal for point-of-care applications due to their portability and affordability.^13,25^ Fluorescence-based assays and surface-enhanced Raman spectroscopy (SERS) provide high selectivity through molecular probes or plasmonic enhancement but often face interference from biological matrices.^26,27^ Emerging techniques, such as aptamer-based biosensors and nanomaterial-enhanced platforms, address challenges like low abundance and matrix complexity, showcasing improved sensitivity and multiplexing capabilities.^28^ Among these, electrochemical methods stand out for their balance of simplicity, cost-effectiveness, and adaptability to wearable or miniaturized devices, aligning with the growing demand for decentralized healthcare monitoring.

Although PLAL has been widely recognized as a sustainable route for producing high-purity nanomaterials, its potential for enabling ligand-free NiNPs with intrinsic electroactive surface chemistry remains underexplored in electrochemical biosensing.^10^ The formation of multiphase Ni/NiO/Ni­(OH)_2_ architectures during ablation in water provides a unique platform that differs fundamentally from chemically synthesized NiNPs, which typically require stabilizers that limit surface accessibility.^5,7^ This synthesis route opens opportunities for developing greener sensing interfaces with improved reproducibility, biocompatibility, and scalability.^29^ Furthermore, PLAL-derived nanostructures hold promise for future applications in integrated microdevices, wearable sensors, and advanced point-of-care technology.

To the best of our knowledge, the electrochemical surface behavior of ligand-free NiNPs synthesized via PLAL remains inadequately explored, particularly for DA detection. While PLAL-generated NiNPs are recognized for their high purity and environmentally friendly synthesis, their electrochemical performance in detecting DA, which is a key biomarker for neurological and psychiatric disorders, has not been systematically studied. By integration of green nanotechnology with advances in electrochemical biosensing, this study presents a novel, sustainable, and efficient strategy leveraging ligand-free, solvent-stabilized NiNPs. This work contributes to the development of greener electrochemical sensing interfaces based on ligand-free nickel-based nanomaterials, advancing next-generation diagnostic tools with improved sensitivity and environmental safety.

## 2 Materials and Methods

### 2.1 Chemicals and Materials

Dopamine hydrochloride (Merck, 98%), Nafion (Sigma-Aldrich, 5%), l-ascorbic acid C_6_H_8_O_6_ (Sigma-Aldrich), histamine dihydrochloride (Thermo Scientific Chemicals, 99%), creatinine (Thermo Scientific Chemicals, 98%), glucose (Sigma-Aldrich, 96%), L-tryptophan (Sigma-Aldrich, 98%), MgCl_2_ (Vetec, 99%), CaCl_2_ (Vetec, 99%), and potassium chloride (Sigma-Aldrich, 99.5%) were of analytical grade. The supporting electrolytes, in the form of three different buffers, were prepared as follows: McIlvaine with disodium phosphate Na_2_HPO_4_ (Merck, 99%) and citric acid H_3_C_6_H_5_O_7_ (Vetec, 99%); B–R with boric acid H_3_BO_3_ (Synth, 99%), acetic acid C_2_H_4_O_2_ (Vetec, 99%) and phosphoric acid H_3_PO_4_ (Vetec, 95%); Glycine-HCl with glycine C_2_H_5_NO_2_ (Merck, 99%) and hydrochloric acid HCl (Merck, 37%). The solution of hexaammineruthenium chloride [Ru­(NH_3_)_6_]­Cl_3_ (III) and hexaammineruthenium chloride [Ru­(NH_3_)_6_]­Cl_2_ (II) (Sigma-Aldrich, >99%) at 1 mmol L^–1^ was used as a redox probe for electrochemical measurements. The solutions were prepared using ultrapure water provided by a Milli-Q system (resistivity ca. 18.2 MΩ cm) manufactured by Millipore (Bedford, USA).

### 2.2 Synthesis of NiNPs

Colloidal suspensions of NiNPs were synthesized via PLAL using ultrapure water as the liquid medium. A rectangular high-purity nickel target (99.9%) was ablated using a Super Mini Fiber Laser system (Translaser, Brazil) controlled by the EzCad2.0 (lite) system (Beijing JCZ Technology Co., Ltd., Beijing, China) operating at its fundamental emission wavelength of 1064 nm. The laser parameters were configured to deliver a power output of 20 W, a spot size of 12 μm, with a pulse width of 110 ns, and a repetition rate of 60 kHz. The target was immersed in a glass vessel filled with 20 mL of ultrapure water, with the liquid level maintained at 7 mm above the target surface. During ablation, continuous magnetic stirring (300 rpm) ensured the homogeneous dispersion of NPs and minimized cavitation-induced scattering of the laser beam. The ablation process was conducted for a fixed duration of 30 min under ambient conditions to ensure reproducibility.

### 2.3 Apparatus

High-resolution transmission electron microscopy (HRTEM) of the NiNPs was carried out using a Hitachi HD-2700 aberration-corrected scanning transmission electron microscope (STEM) operating at an acceleration voltage of 200 kV. For sample preparation, 10 μL of the colloidal suspension was deposited onto carbon-coated copper grids and allowed to air-dry prior to imaging.

X-ray diffraction (XRD) analysis was performed using a Rigaku MiniFlex600 diffractometer operated at 40 kV and 15 mA to investigate the crystalline structure of the NiNPs. Data acquisition was carried out with a step size of 0.05° and a scanning rate of 2.5° min^–1^ over a 2θ range of 20° to 90°. Sample preparation involved centrifugation of 1000 μL aliquots at 6000 rpm for 20 minutes. Following centrifugation, the supernatant was discarded, and the concentrated NiNPs were deposited onto a glass substrate and dried in an oven at 40 °C. This procedure was repeated until approximately 100 mg of dry material was obtained for analysis.

X-ray photoelectron spectroscopy (XPS) analysis was performed using a Thermo Scientific K-α spectrometer equipped with a monochromatic Al Kα X-ray source (*h*ν = 1486.6 eV) and a 400 μm spot size. Measurements were conducted under ultrahigh vacuum conditions (base pressure <10^–8^ mbar), with charge neutralization applied using a flood gun. Survey spectra were acquired with a pass energy of 200 eV, step size of 1 eV, dwell time of 10 ms, and 10 scans per sample. High-resolution spectra were collected for the Ni 2p and O 1s core levels using a pass energy of 20 eV, step size of 0.025 eV, dwell time of 100 ms, and 10 scans. All spectra were calibrated using the adventitious carbon C 1s peak at 284.8 eV as reference. Data processing and peak deconvolution were performed using Thermo Scientific Advantage software, employing mixed Gaussian–Lorentzian functions for fitting and background subtraction.

Raman spectroscopy measurements were performed to investigate the vibrational properties and local structural features of the NiNPs. The spectra were acquired using a WITec Alpha300R confocal Raman spectrometer equipped with a 532 nm excitation laser. Data were collected over the spectral range of 250–1500 cm^–1^. The measurements were carried out under ambient conditions, and the laser was focused onto the sample using a confocal optical configuration to ensure high spatial resolution and minimize background interference. The collected Raman signal was processed using the instrument’s proprietary software, including baseline correction and spectral deconvolution when necessary, to enable accurate identification of vibrational modes.

Dynamic light scattering (DLS) measurements were conducted using a Malvern Zetasizer Nano ZS, utilizing a 633 nm He–Ne laser source. Data were acquired at a backscattering angle of 173° under controlled temperature conditions (25 °C), with water (refractive index *n* = 1.33) as the dispersing medium. Zeta potential (ζ) was also determined using the same instrument, with 750 μL of each sample loaded into disposable folded capillary cells for analysis.

### 2.4 Electrochemical Measurements

Electrochemical measurements were performed using a μStat-I 400s potentiostat (Metrohm DropSens, Oviedo, Spain), with data acquisition and control managed via DropView 800 software. A conventional three-electrode configuration was employed, consisting of a GCE (Ø = 3 mm, ALS, Japan) as the working electrode, a saturated Ag/AgCl, KCl_(sat)_ reference electrode, and a platinum wire serving as the counter electrode. All experiments were conducted in a 10 mL electrochemical cell at ambient temperature.

The GCE surface was sequentially polished using alumina slurries (0.3 μm followed by 0.05 μm particle size), rinsed exhaustively with distilled water, and ultrasonicated in a 1:1 (v/v) ethanol/water solution for 15 min to remove residual contaminants and used for modification. For electrode surface modification, a homogeneous nanocomposite dispersion was prepared by mixing 97 μL of the NiNPs suspension (0.44 mg mL^–1^) with 3 μL of a 5% (w/v) Nafion solution, yielding a final composite containing 0.427 mg mL^–1^ NiNPs and 0.15% (w/v) Nafion. The NiNPs suspension concentration was initially determined based on the total mass of nickel ablated during synthesis and subsequently confirmed by flame atomic absorption spectroscopy. The mixture was then subjected to bath ultrasonication for 30 min to promote colloidal stability and ensure uniform particle distribution. The functionalization protocol involved drop-casting 7 μL of the homogenized NiNPs–Nafion suspension onto the pretreated GCE surface, corresponding to a deposited mass of approximately 3.0 μg of NiNPs and 10.5 μg of Nafion per electrode, as this volume provided full electrode coverage with a uniform film while avoiding cracks or excessive thickness, followed by thermal drying at 60 °C for 10 min to achieve complete solvent evaporation. This protocol was repeated for each modification.

The sensor was characterized using a 1 mmol L^–1^ solution of [Ru­(NH_3_)_6_]­Cl_3_ (III) and [Ru­(NH_3_)_6_]­Cl_2_ (II) with a 0.1 mol L^–1^ KCl solution as redox probe. CV was applied over a potential range from +0.2 V to −0.6 V (vs Ag/AgCl, KCl­(_sat)_) by using a scan rate (ν) of 50 mV s^–1^. EIS measurements were performed at −0.15 V as *V*_ref_ and 100 mV­(rms) as Eamp, in a frequency range of 1.0 × 10^5^ to 0.1 Hz with 100 points.

Electrochemical investigations were conducted across a pH range of 3.0 to 7.0 to assess the influence of proton concentration on reaction dynamics. To evaluate kinetic processes and underlying mechanisms, the scan rate was systematically varied from 10 to 150 mV·s^–1^.

### 2.5 DA Electrochemical Sensing and Preliminary Sample Analyses

For DA quantification, CV was employed to evaluate the system’s response. A calibration curve was constructed (*n* = 3) by successive additions of DA stock solution (0.01 mol·L^–1^) to the B–R supporting electrolyte (0.1 mol L^–1^, pH 3.0). The limits of detection (LOD) and quantification (LOQ) were established according to the International Union of Pure and Applied Chemistry (IUPAC) guidelines.^30^ The following equations were used: LOD = 3.3 s/a and LOQ = 10 s/a, where s is the standard deviation of the intercept and a is the slope of the calibration curve. The sensors were constructed independently for the plots of the calibration curves, renewing the electrode surface before each measurement.

To verify the application of the novel electrochemical method for DA sensing purposes, its response was tested in simulated urine (*n* = 3). Simulated urine samples were prepared following established procedures.^31^ This methodology is commonly reported in the literature for preliminary evaluations.^32^ The simulated urine was diluted 1:50 in B–R buffer (0.1 mol L^–1^, pH 3.0). DA was added in three levels of concentration (*n* = 3) within the method’s linear range to evaluate the recovery response.

To evaluate the method under practical conditions, the performance of the GCE/Nafion/NiNPs electrode toward DA was examined. The precision of the approach was tested through both intraday and interday repeatability studies. For intraday assessment, five consecutive measurements were conducted within a single day, whereas interday reproducibility was determined by recording one measurement per day over five successive days, under the same experimental parameters. The relative standard deviation (RSD) was determined using the formula %RSD = (*s* × 100)/*x*, where *s* represents the standard deviation and *x* denotes the mean response. The method’s selectivity and robustness were further validated by introducing possible interfering substances, including histamine, creatinine, L-tryptophan, glucose, ascorbic acid, MgCl_2_, and CaCl_2_, at a concentration ratio of 1:10 (0.5 μmol L^–1^ DA to 5.0 μmol L^–1^ interferent). The effect of each interfering agent was assessed by comparing the electrode’s peak current responses in the presence and absence of the interfering species.

## 3 Results and Discussion

### 3.1 Material Characterizations

NiNPs were synthesized using PLAL, a surfactant-free and environmentally friendly method that produces colloidal NPs with clean surfaces and well-defined morphologies.^33^ This green synthesis route avoids the use of chemical reducing agents or stabilizers and allows for in situ surface modification via interaction with the surrounding aqueous medium, enabling the formation of complex surface chemistries that are favorable for electrochemical applications.^34^

Figure 1a presents the TEM image along with the particle size distribution histogram of the synthesized NiNPs. The particles exhibit a predominantly quasi-spherical morphology with moderate size dispersion and slight aggregation, even after sonication. The size distribution analysis, based on a statistical evaluation of the TEM images, revealed an average particle diameter of 19.3 ± 8.2 nm. Prior to grid preparation, the sample was sonicated for 30 min to reduce agglomeration effects. Figure 1b shows the corresponding HRTEM image, where lattice fringes can be observed, confirming the crystalline nature of the NPs. Although no clear core–shell structure is evident from the contrast, the presence of oxidized surface species cannot be ruled out and was further investigated using complementary characterization techniques.

**Figure 1:**
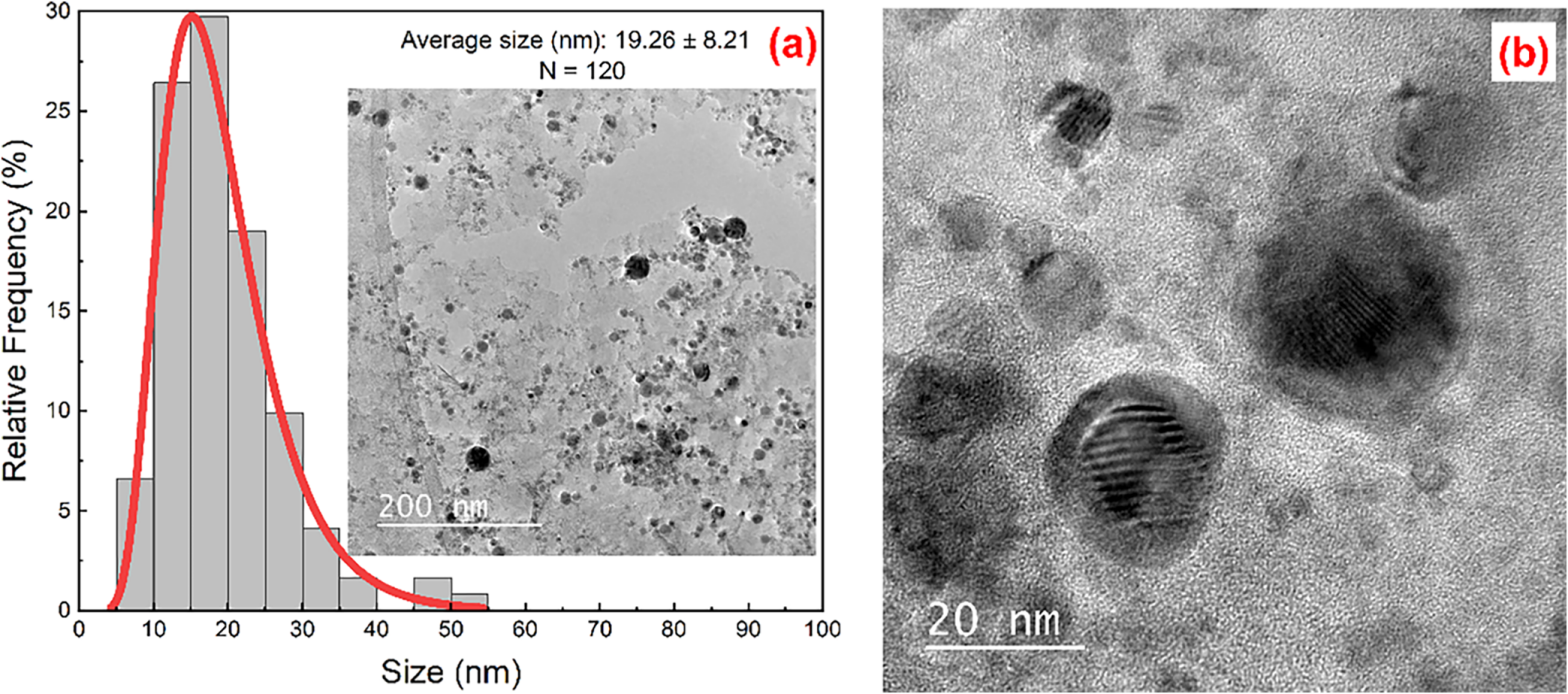
(a) TEM image of NiNPs and corresponding particle size distribution histogram with Gaussian fitting, showing a predominantly quasi-spherical morphology with moderate size dispersion and an average diameter of 19.3 ± 8.2 nm. (b) HRTEM image of an individual nanoparticle, revealing visible lattice fringes that confirm the crystalline nature of the material.

XRD analysis of the oven-dried powder (Figure 2a) revealed a multiphase composition. Distinct peaks at 44.40°, 51.75°, and 76.25° were assigned to the (111), (200), and (220) planes of face-centered cubic (fcc) metallic nickel (JCPDS no. 00–004–0850). In addition, peaks at 37.20°, 43.25°, and 62.90° correspond to NiO (JCPDS no. 00–047–1049), while reflections at 33.10°, 38.15°, and 59.05° were indexed to Ni­(OH)_2_ (JCPDS no. 00–001–1047). The coexistence of these phases is attributed to surface oxidation and hydroxylation occurring during the PLAL process in water.^34,35^ No thermal annealing or postsynthesis treatment was applied, confirming that the oxide/hydroxide layers formed intrinsically during laser ablation in the aqueous environment.

**Figure 2:**
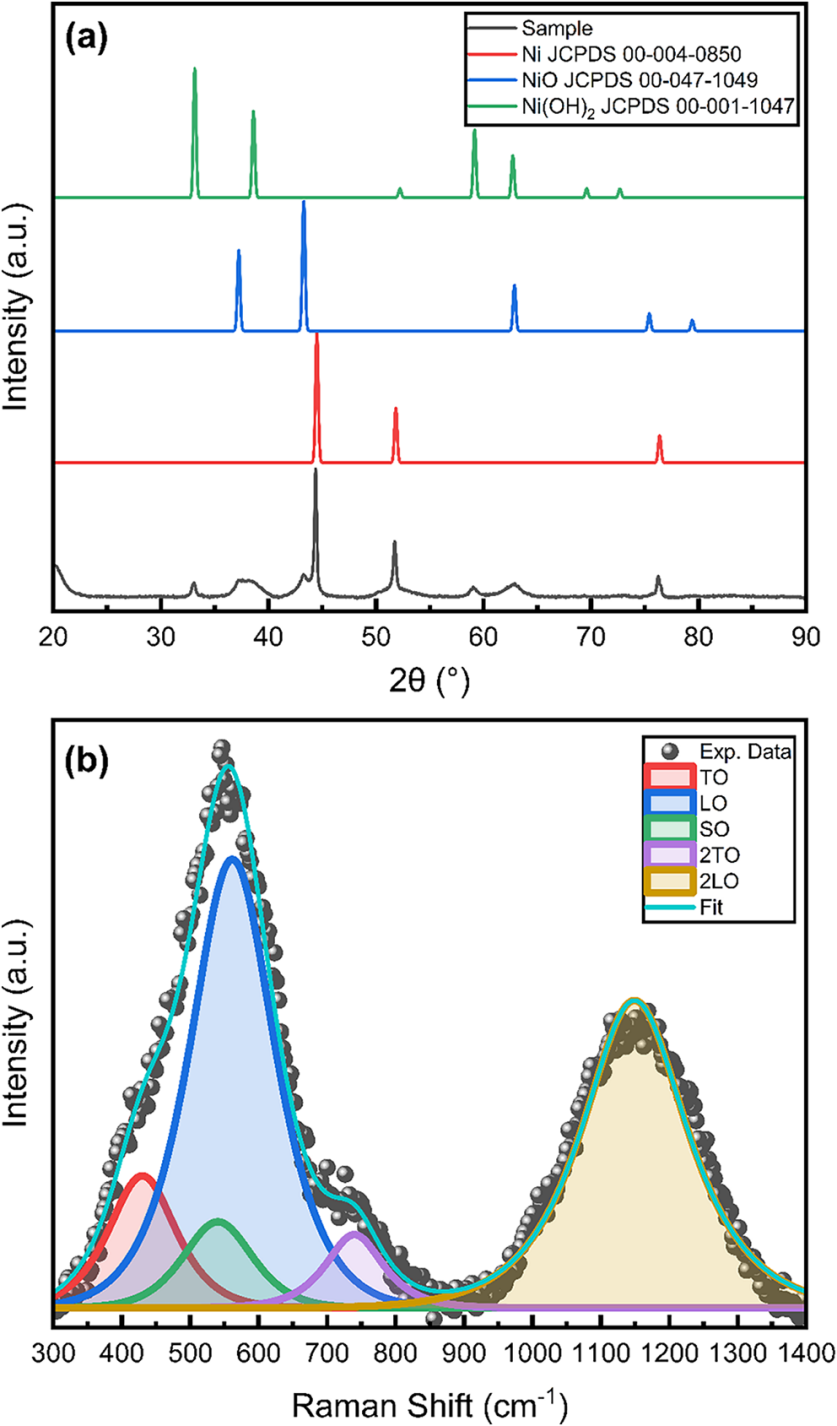
(a) XRD pattern of NiNPs synthesized by PLAL, showing peaks indexed to fcc Ni, NiO, and Ni­(OH)_2_, indicating a multiphase composition arising from surface oxidation and hydroxylation. (b) Raman spectrum with deconvolution, displaying the dominant LO mode of NiO (∼561.7 cm^–1^), along with TO, SO, and second-order (2TO and 2LO) modes. Band broadening reflects phonon confinement and structural disorder in nanocrystalline NiO.

To further quantify the phase composition, Rietveld refinement was performed on the XRD pattern (Figure S1, Supporting Information), allowing a semiquantitative evaluation of the multiphase system. The refinement results indicate that the sample is predominantly composed of nickel hydroxide with a phase distribution of Ni:Ni­(OH)_2_:NiO = 3.33(9):93.4(2):3.3(1), confirming that Ni­(OH)_2_ is the predominant crystalline phase. In contrast, both metallic Ni and NiO are present as only minor components. The predominance of Ni­(OH)_2_ is fully consistent with the aqueous PLAL synthesis conditions, which favor extensive surface hydroxylation and the stabilization of Ni^2+^ species in hydroxide environments. This result also reinforces the XPS findings, where hydroxyl-related species dominate surface chemistry. The relatively low fraction of NiO and metallic Ni suggests that these phases are either confined to the nanoparticle core or present as poorly crystalline domains.^36,37^

Raman spectroscopy was employed to further investigate the vibrational properties and local structural features of the NiNPs, with particular sensitivity to oxide phases and defect-related effects. The full Raman spectrum acquired over a wide spectral range is presented in the Supporting Information (Figure S2). Based on this complete data set, the spectral region of interest was selected for detailed analysis and deconvolution, as shown in Figure 2b. The Raman spectrum exhibits a dominant broad band centered at approximately 561.7 cm^–1^, which is assigned to the longitudinal optical (LO) phonon mode of NiO. The pronounced broadening of this band (FWHM ≈ 144.6 cm^–1^) is indicative of phonon confinement effects and structural disorder, both of which are characteristic of nanocrystalline materials with reduced grain size. In addition to the LO mode, a shoulder in the 500–540 cm^–1^ region, centered at approximately 540.4 cm^–1^ with a FWHM of about 119.5 cm^–1^, is attributed to surface optical (SO) phonon modes. These modes arise from symmetry breaking at the nanoparticle surface and become increasingly prominent as the surface-to-volume ratio increases. The partial overlap between LO and SO contributions is consistent with nanoscale NiO systems, where surface disorder leads to significant band broadening and prevents the resolution of well-defined individual peaks.^38,39^

A weaker band centered at approximately 429.9 cm^–1^ (FWHM ≈ 115.6 cm^–1^) is assigned to the transverse optical (TO) mode, which becomes Raman-active due to defect-induced relaxation of selection rules. Furthermore, a broad feature observed at around 1149.1 cm^–1^ (FWHM ≈ 180 cm^–1^) is associated with the second-order longitudinal optical (2LO) mode, confirming the presence of multiphonon scattering processes. An additional second-order contribution (2TO) is also identified near 739.8 cm^–1^ with a comparatively narrower width (FWHM ≈ 99.3 cm^–1^).^38^

Because of the significant overlap of vibrational modes, peak deconvolution was performed to achieve a more accurate interpretation of the spectral features, allowing the separation of contributions from TO, LO, SO, and higher-order modes. The analysis indicates that the LO mode provides the most intense contribution to the overall Raman response, followed by the 2LO band, while the TO, SO, and 2TO modes exhibit comparatively lower intensities. These relative contributions reflect the vibrational response of the system and should not be interpreted as a direct measure of the phase composition. The relatively large FWHM values observed for all vibrational modes further corroborate the presence of structural disorder and phonon confinement effects typical of nanocrystalline NiO.

Overall, the Raman spectral profile is consistent with defect-rich, nanostructured NiO formed on the surface of the NiNPs, corroborating the XRD results and reinforcing the presence of oxide layers generated during the PLAL process.

The surface chemical composition and oxidation states of the NiNPs synthesized were investigated by XPS, with particular emphasis on the Ni 2p and O 1s core-level regions (Figure 3a,b). A survey spectrum (Figure S3) confirms the elemental composition of the samples, showing the presence of Ni and O as the main constituents.^40,41^

**Figure 3:**
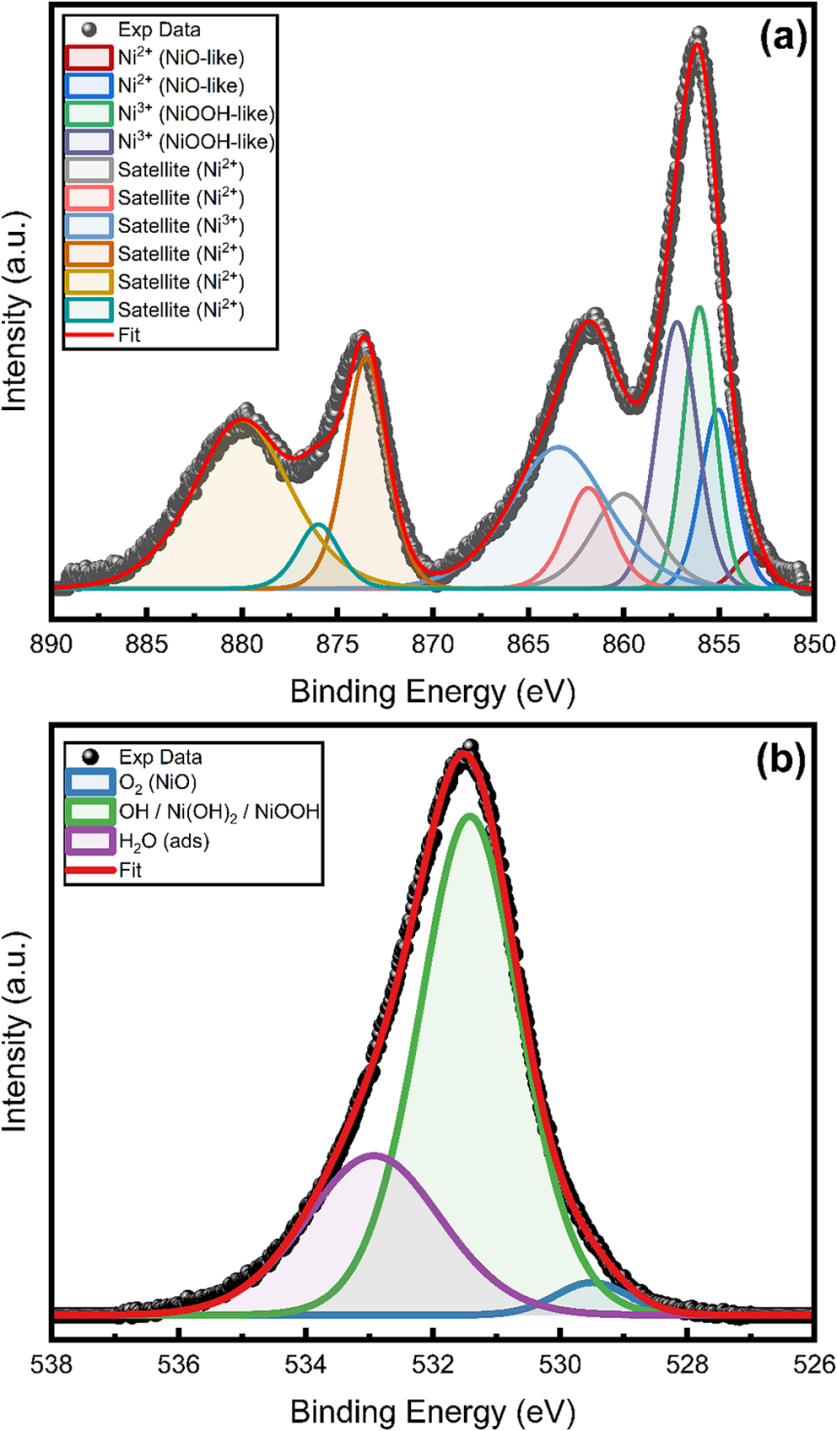
High-resolution XPS spectra of NiNPs synthesized by PLAL. (a) Ni 2p spectrum showing contributions from Ni^2+^ (oxide/hydroxide) and Ni^3+^ species, along with characteristic shakeup satellites, indicating predominantly oxidized nickel. (b) O 1s spectrum deconvoluted into lattice oxygen, hydroxyl groups, and adsorbed water, revealing a hydroxyl-rich surface consistent with the formation of Ni­(OH)_2_/NiOOH species.

The high-resolution Ni 2p spectrum (Figure 3a) exhibits the characteristic spin–orbit doublet corresponding to the Ni 2p_3/2_ and Ni 2p_1/2_ components, accompanied by well-defined shakeup satellite features, indicative of the complex electronic structure typically associated with oxidized nickel species.^40,41^ The deconvolution of the Ni 2p_3/2_ region reveals multiple contributions centered at approximately 853.3, 855.0, 856.0, and 857.2 eV. The component at ∼853.3 eV is attributed to Ni^2+^ species in an oxide-like environment (NiO) and/or partially reduced nickel species, while the contribution at ∼855.0 eV is consistent with Ni^2+^ in NiO-like coordination.^40,42^ The peak at ∼856.0 eV is assigned to Ni^2+^ species in hydroxide environments (Ni­(OH)_2_), whereas the higher binding energy component at ∼857.2 eV is attributed to Ni^3+^ species, commonly associated with oxyhydroxide phases such as NiOOH.^40,43^ The presence of intense shakeup satellites in the 861–865 eV region further supports the predominance of Ni^2+^ species with strong electron correlation effects.^40,41^ The detailed fitting parameters used for the Ni 2p region, including binding energies, full width at half-maximum (FWHM), and relative peak areas, are provided in Table S1.

No distinct contribution is observed at ∼852.6 eV, indicating the absence of metallic Ni^0^ at the nanoparticle surface within the detection limits of XPS.^42^ Nevertheless, the presence of a minor low-binding-energy component (∼853.3 eV) suggests contributions from oxide-like Ni^2+^ species rather than fully metallic nickel.

Given the well-known complexity of Ni 2p spectra, arising from multiplet splitting, shakeup processes, and overlapping contributions from different oxidation states, quantitative chemical state analysis must be interpreted with caution.^40,41^ In this context, a semiquantitative evaluation of the Ni 2p_3/2_ envelope was performed by considering only the main peak components and excluding satellite contributions, following common practices reported in the literature.^40^

This analysis indicates that Ni^2+^ species, encompassing both oxide- and hydroxide-like environments, represent the dominant surface contribution, while Ni^3+^ species constitute a substantial but comparatively lower fraction. The estimated relative distribution between oxidation states is approximately ∼60% Ni^2+^ and ∼40% Ni^3+^. It should be emphasized that these values are approximate and intended to provide a comparative indication of the relative abundance of oxidation states, rather than an absolute quantification, due to the intrinsic limitations associated with XPS analysis of nickel-based systems.^40^

Complementary information is obtained from the O 1s spectrum (Figure 3b), which was deconvoluted into three main components located at approximately 529.5, 531.4, and 532.9 eV. These contributions are attributed to lattice oxygen (O^2–^) in NiO, hydroxyl groups associated with Ni­(OH)_2_/NiOOH species, and adsorbed molecular water, respectively.^44^ Quantitative analysis reveals that hydroxyl-related species dominate the surface composition (∼68%), followed by adsorbed water (∼29%), while lattice oxygen accounts for only a minor fraction (∼3%). The detailed fitting results for the O 1s region are summarized in Table S2. This low contribution of O^2–^ indicates that bulk-like NiO is not the dominant surface phase, although it may still be present in subsurface or core regions.

A strong correlation between the Ni 2p and the O 1s spectra provides consistent evidence for the proposed surface chemistry. In particular, the predominance of hydroxyl species in the O 1s spectrum is in good agreement with the significant presence of Ni^2+^ (hydroxide) and Ni^3+^ (oxyhydroxide) species in the Ni 2p region, as Ni­(OH)_2_ and NiOOH are intrinsically associated with hydroxylated environments.^40^ Likewise, the relatively low contribution of lattice oxygen is consistent with the limited presence of oxide-like Ni^2+^ species. The substantial fraction of adsorbed water further indicates a highly hydrated surface, as expected for NPs synthesized in aqueous media.

This surface composition can be rationalized considering the synthesis conditions. During pulsed laser ablation in water, ablated Ni species are rapidly quenched in a highly reactive liquid environment, leading initially to the formation of Ni^2+^ species, followed by further oxidation and extensive hydroxylation at the nanoparticle interface.^34,45^ As a result, the NPs likely exhibit a core–shell-like structure, in which the inner core may retain metallic or NiO-like characteristics, while the outer surface is dominated by Ni­(OH)_2_/NiOOH species and adsorbed water. Such structures are widely reported for NPs synthesized by laser ablation in liquids and are often associated with enhanced surface reactivity.^36,46^

Overall, the XPS results demonstrate that the NiNPs produced in ultrapure water possess a highly oxidized and hydroxylated surface, predominantly composed of Ni^2+^ species, with a significant contribution from Ni^3+^ oxyhydroxide phases. The semiquantitative agreement between the Ni 2p and O 1s analyses, supported by the survey spectrum (Figure S3) and detailed fitting parameters (Tables S1 and S2), provides robust evidence for a hydroxyl-rich surface chemistry, which is expected to play a key role in the physicochemical and functional properties of the material.^40^

DLS results are presented in Figure S4a, showing a narrow size distribution centered at 26.9 ± 2.2 nm. This value is larger than the average size observed in HRTEM due to the hydrodynamic nature of the DLS measurement, which includes the solvation layer and any loosely adsorbed species in the colloidal state. This discrepancy is expected, as DLS measures particles in their native dispersed form, while HRTEM images dried particles under high vacuum.^47^ Together, these results indicate that the NiNPs exhibit reasonably good size homogeneity, although minor polydispersity and aggregation cannot be excluded.

Zeta-potential measurements, shown in Figure S4b, were conducted with no further treatments after synthesis. The as-prepared NiNPs exhibited a surface potential of +24.4 mV, suggesting moderate electrostatic stabilization.^48^ This behavior may be associated with surface defects such as a reduced density of hydroxyl groups generated during the highly energetic PLAL process. These defects lead to a positive ζ-potential, which accounts for the observed positive values and is consistent with a previous report.^49^

However, slight sedimentation was observed over time, indicating that additional stabilization would be beneficial for long-term storage or device integration. To improve colloidal stability and facilitate sensor fabrication, the NPs were mixed with Nafion, a sulfonated tetrafluoroethylene-based polymer commonly used in electrochemical sensors.^50,51^ Upon incorporation into the Nafion matrix, the surface charge of the resulting Nafion/NiNPs composite shifted to −25.13 mV, consistent with strong electrostatic interactions between the negatively charged sulfonate groups of Nafion and the positively charged NiNPs surfaces.

The combined structural and surface analyses provide a consistent and comprehensive picture of the synthesized NiNPs as a multiphase system strongly dominated by nickel hydroxide rather than a simple metallic Ni core with a thin oxide shell. XRD analysis, supported by Rietveld refinement, reveals that Ni­(OH)_2_ is the predominant crystalline phase, while metallic Ni and NiO are present as only minor components. Raman spectroscopy further confirms the presence of defect-rich nanostructured NiO, and XPS demonstrates that the nanoparticle surface is highly oxidized and hydroxylated, being primarily composed of Ni^2+^ and Ni^3+^ species in hydroxide and oxyhydroxide environments along with a significant contribution from hydroxyl groups and adsorbed water. These results indicate that oxidation and hydroxylation are not minor surface effects but instead define the physicochemical nature of NPs synthesized by PLAL in aqueous media.

From a functional perspective, this hydroxide-rich composition may be advantageous, as Ni­(OH)_2_/NiOOH phases provide electroactive Ni­(II)/Ni­(III) redox couples that have been widely associated with facilitated charge-transfer processes and improved electrocatalytic activity in a range of electrochemical systems.^52–54^ In this context, the coexistence of minor metallic Ni domains may contribute to electrical conductivity, while the oxidized phases govern the catalytic response, resulting in a synergistic system. Therefore, the observed oxidation state distribution should be regarded as an intrinsic and beneficial characteristic of the material rather than a limitation.

### 3.2 Electrochemical Characterization

NiNPs are widely recognized for their catalytic versatility, particularly in electrochemical applications.^34^ Despite the high purity and promising electrochemical properties of laser-synthesized NiNPs, their use in sensor development has remained limited. In this study, NiNPs were employed for the electrochemical detection of DA. To prepare the sensing platform, the NPs were drop-cast onto a GCE, and a small amount of Nafion was incorporated to enhance the film stability and prevent nanoparticle leaching from the electrode surface.

The electrochemical behavior of the modified electrodes was evaluated using CV in DA solution and EIS in a [Ru­(NH_3_)_6_]^3+/2+^ electrolyte, as shown in Figure 4a–b. CV measurements were carried out in 70 μM DA prepared in 0.1 mol L^–1^ B–R buffer (pH 7.0) at a scan rate of 50 mV s^–1^. The unmodified GCE displayed redox processes at approximately +0.57 V and +0.30 V, with cathodic (Ipc) and anodic (Ipa) peak currents of 1.43 ± 0.003 μA and −0.86 ± 0.006 μA, respectively. Upon modification with NiNPs (GCE/NiNPs), both peak positions and current intensities shifted slightly, with Ipc at +0.63 V (1.32 ± 0.041 μA) and Ipa at +0.25 V (−0.70 ± 0.004 μA), indicating limited interaction between the NiNPs and the electrode surface. In contrast, the GCE/Nafion/NiNPs exhibited a markedly enhanced redox response, with Ipc of 16.93 ± 1.594 μA at +0.54 V and Ipa of −14.12 ± 1.127 μA at +0.26 V. These values correspond to approximately 12- and 16-fold increases in current intensity response, respectively, compared to the bare GCE. This pronounced enhancement indicates a synergistic effect in which Nafion promotes electrostatic preconcentration of DA, while the ligand-free NiNPs provide electroactive Ni-based surface sites that mediate DA oxidation.

**Figure 4:**
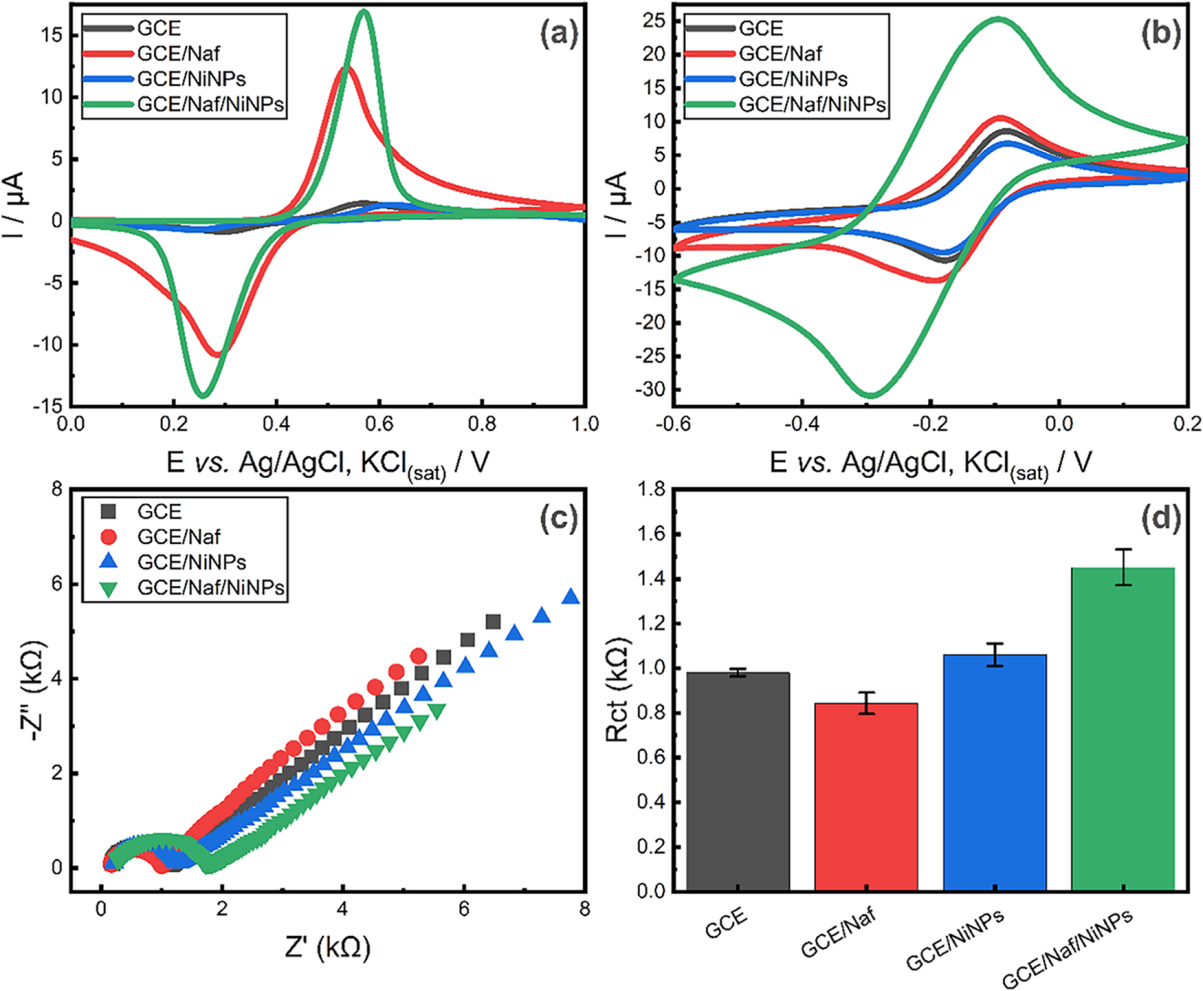
Electrochemical evaluation of NiNPs for DA sensing and related characterizations: (a) voltammetric response for 70 μM DA using GCE, GCE/Nafion, GCE/NiNPs, and GCE/Nafion/NiNPs in B–R buffer at pH 7.0, (b) CV profiles of [Ru­(NH_3_)_6_]­Cl_3_ (Ru^3+^) and [Ru­(NH_3_)_6_]­Cl_2_ (Ru^2+^) in 0.1 mol L^–1^ KCl, (c) EIS using the same redox probes in 0.1 mol L^–1^ KCl, and (d) charge-transfer resistance (*R*_ct_) values derived from the Randles equivalent circuit.

The improved sensitivity of the Nafion/NiNPs composite for DA detection arises from well-defined electrostatic and electrochemical interactions. The combination of laser-ablated NiNPs with the polymer Nafion yielded the highest performance, reaching a cathodic peak current of 16.93 ± 1.594 μA. This synergistic effect is attributed to a substantial shift in surface charge, where the zeta potential changed from +24.4 mV (for bare NiNPs) to −25.13 mV (Figure S4b) after incorporation into the Nafion matrix (Nafion/NiNPs). This charge reversal critically enhances the electrostatic attraction between the negatively charged sulfonate groups (−SO_3_^–^) of Nafion and the protonated form of DA (positively charged at pH 7.0), while simultaneously reducing electrostatic repulsion between the NiNPs and DA. Additionally, the optimized charge environment promotes stronger interactions between NiNPs and DA, facilitating oxidative catalytic activity that contributes to selective binding and signal amplification.^29,55^ These findings highlight the importance of tailoring the surface charge of NiNPs to complement Nafion’s ion-exchange characteristics, ultimately enhancing the electrochemical response.

The charge-selective behavior of the Nafion/NiNPs composite was further investigated using [Ru­(NH_3_)_6_]^3+/2+^, a cationic redox probe. As illustrated in Figure 4c,d, EIS revealed a marked increase in charge-transfer resistance (*R*_ct_) for the GCE/Nafion/NiNPs compared to the unmodified GCE. This increase in *R*_ct_ is likely due to the accumulation and partial retention of cationic species within the nanocomposite film, driven by electrostatic interactions. Nafion, possessing negatively charged sulfonate groups, has a strong affinity for cations, which may restrict their diffusion to the electrode surface.^29,56^ Nevertheless, the elevated peak current intensities observed in CV suggest that the high surface area and electrocatalytic properties of NiNPs facilitate enhanced electron transfer once the initial charge barrier is overcome. Additionally, Nafion’s ion-exchange capacity may locally concentrate the redox probe near the electrode, effectively increasing its availability for electrochemical reactions. The observed shifts and enhanced electrochemical response in the CV likely result from altered electron-transfer kinetics and diffusion constraints introduced by the composite layer.^24,57^

It should be noted that the increased charge-transfer resistance (*R*_ct_) and the relatively large peak-to-peak separation (Δ*E*_p_ ≈ 400 mV) observed for the GCE/Nafion/NiNPs using the [Ru­(NH_3_)_6_]^3+/2+^ redox probe are characteristic of a quasi-reversible outer-sphere electron-transfer process.^56,58^ This behavior arises primarily from electrostatic and ion-transport limitations imposed by the Nafion matrix, which acts as a cation-exchange membrane and can partially hinder or modulate the access of positively charged redox species to the electrode surface. As a result, larger Δ*E*_p_ values are commonly observed for cationic outer-sphere probes at GCE/Nafion/NiNPs, despite preserved electronic conductivity of the underlying electrode.^56,58^

This electrochemical response contrasts with that observed for DA detection, where the sensing mechanism is governed by electrostatic preconcentration of protonated DA within the Nafion matrix and by electrocatalytic mediation at NiO/Ni­(OH)_2_ surface sites.^24,59,60^ In this case, analyte-specific interactions and surface redox activity dominate the electron-transfer process, leading to enhanced voltammetric responses. Therefore, the large Δ*E*_p_ observed for the [Ru­(NH_3_)_6_]^3+/2+^ probe reflects charge-selective interfacial effects rather than limitations in the intrinsic electrochemical performance of the GCE/Nafion/NiNPs.

The impedance data confirmed the presence of a resistive element at high frequencies and a diffusion-related component at low frequencies, consistent with the behavior of a charge-selective interface. This response reflects the function of Nafion as both a cation-selective membrane that promotes the accumulation of DA and a barrier that restricts the access of interfering species. Such dual selectivity reinforces the potential of the sensing platform for applications in complex sample matrices.

While this analysis provides useful insights into the interfacial behavior, some limitations regarding equivalent circuit modeling should be considered. Although a Randles-type equivalent circuit was employed to estimate the charge-transfer resistance (*R*_ct_) for comparative purposes, the applicability of more detailed equivalent circuit modeling was carefully evaluated. Attempts to fit the impedance spectra using more complex circuits resulted in poor statistical quality and significant parameter dispersion, indicating nonunique and physically unreliable solutions. This behavior is attributed to the nonideal and distributed nature of the Nafion/NiNP interface, involving surface heterogeneity and adsorption processes. Therefore, the analysis was limited to a qualitative and comparative interpretation of the impedance data, which is sufficient to support the conclusions of this study.

Despite these limitations, the overall impedance behavior provides consistent insight into the interfacial properties of the system. The Nafion/NiNPs composite integrates the high purity of laser-ablated NPs with controlled surface charge, demonstrating how interfacial modification can address conventional challenges in electrochemical detection. This design strategy provides a promising foundation for the selective and sensitive monitoring of neurochemicals under real-world conditions.

In comparison to NiNPs synthesized through conventional chemical routes, those produced by PLAL offer notable benefits in terms of purity, surface accessibility, and environmental compatibility. Chemical synthesis methods often involve surfactants or reducing agents that may adsorb onto the nanoparticle surface, potentially blocking active sites and diminishing the electrocatalytic efficiency. In contrast, NiNPs obtained via PLAL are generated without the use of stabilizing ligands, resulting in clean, highly reactive surfaces. This absence of surface contaminants enhances electron-transfer kinetics and supports more efficient electrochemical interactions, positioning laser-synthesized NiNPs as a promising alternative for sensor applications.

### 3.3 Evaluation of the Supporting Electrolyte and Mass Transfer Regime

To investigate how pH influences the electrochemical behavior of DA and propose a mechanism explaining the processes occurring on the surface of the GCE/Nafion/NiNPs sensor, CV was employed. The study was conducted in a 0.1 mol L^–1^ B–R buffer solution, with pH varying from 3.0 to 7.0, at a scan rate of 50 mV s^–1^. The results showed that the anodic (Epa) and cathodic (Epc) peak potentials are pH-dependent, shifting to more negative values as the pH increases (Figure S5a–c). This shift indicates the involvement of protons in the oxidation and reduction reactions of DA.^24^ Additionally, it was observed that the highest peak current intensity was achieved at pH 3.0, i.e., in an acidic medium. For this reason, this pH value was selected for subsequent optimization steps of the system.

Previous studies have demonstrated that the choice of supporting electrolyte significantly affects proton transport and the resulting electrochemical response across various buffering systems.^13,24,61,62^ To explore this influence, different buffers, including B–R, McIlvaine, and Glycine-HCl (Glyc-HCl), were evaluated. All buffers were prepared at a concentration of 0.1 mol L^–1^ and adjusted to pH 3.0. The electrochemical behavior was assessed using CV in the presence of 50 μmol L^–1^ DA. As illustrated in Figure S6a–b, the Glyc-HCl buffer produced the highest Ipa. However, upon analyzing the peak shape, the B–R buffer exhibited a superior balance between analytical response and peak width, enhancing the method’s selectivity. Consequently, B–R was selected for further analysis. Subsequent investigations focused on the effect of B–R buffer concentration, ranging from 0.025 to 0.3 mol L^–1^ (Figure S6c–d). Although the highest peak current was recorded at 0.3 mol L^–1^, the 0.1 mol L^–1^ concentration provided a more favorable compromise by maintaining a strong current response while delivering a narrower peak. This improved resolution supports better discrimination against potential interfering species, leading to the adoption of 0.1 mol L^–1^ B–R buffer for the remaining experiments.

The analysis of scan rate variation is essential for elucidating the reaction mechanism and the sensor’s functionality, as well as for optimizing DA detection conditions. Figure 5a displays the cyclic voltammograms for a 50 μmol L^–1^ DA concentration in a 0.1 mol L^–1^ B–R (pH 3.0), using the GCE/Nafion/NiNPs sensor, with scan rates ranging from 10 to 150 mV s^–1^. Increasing the scan rate led to an increase in peak current and a broadening of the separation between *E*_pa_ and *E*_pc_, a behavior typical of *quasi*-reversible systems.^24,63^ In reversible diffusion-controlled systems, peak currents scale with *v* ^1/2^ and peak potentials remain nearly constant. In irreversible or quasi-reversible systems, peak potentials shift with scan rate, leading to increased peak separation.^64^ The increase in peak separation with scan rate indicates quasi-reversible electron-transfer behavior.

**Figure 5:**
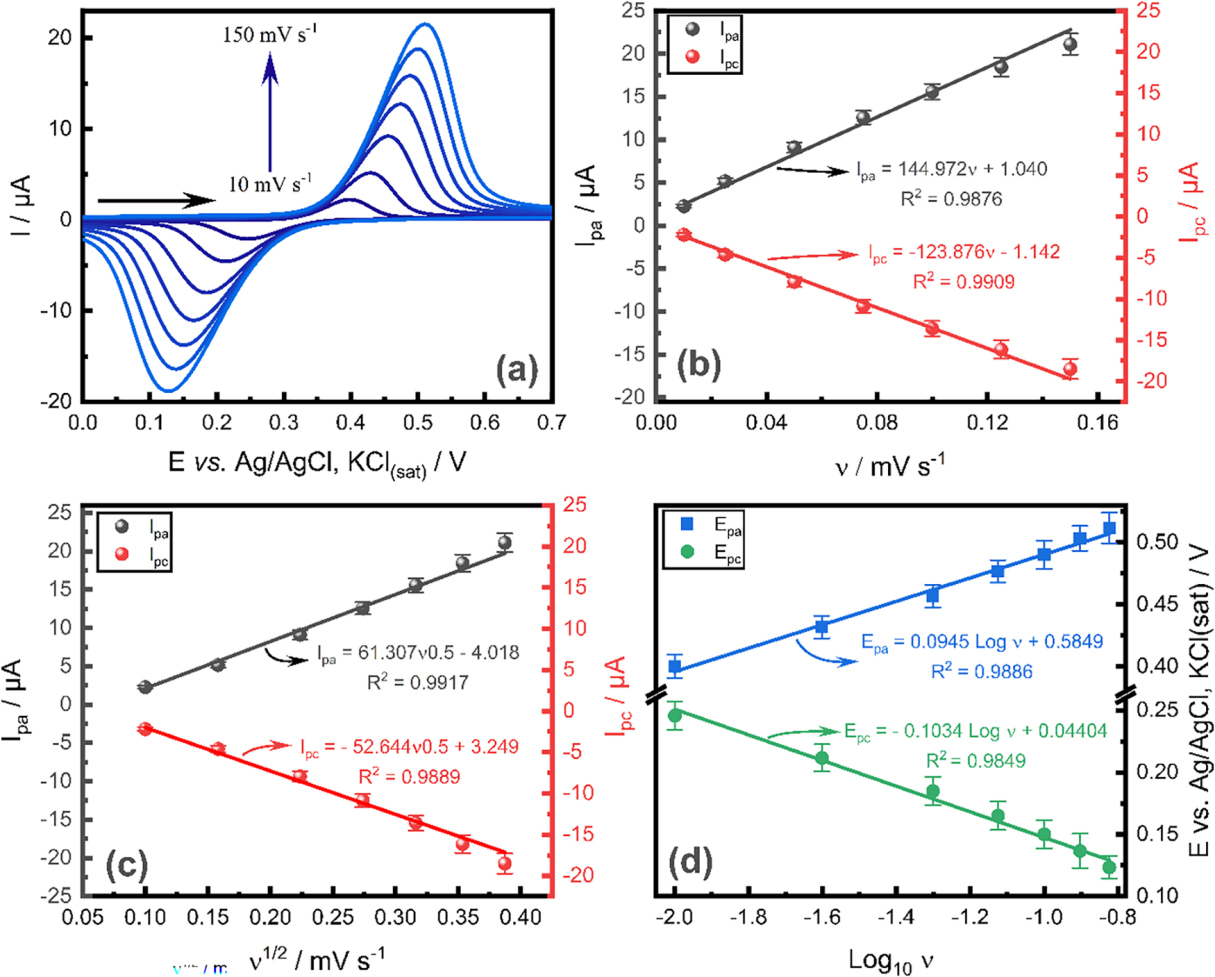
Effect of scan rate on the electrochemical response of DA (50 μmol L^–1^) at GCE/Nafion/NiNPs in 0.1 mol L^–1^ B–R buffer (pH 3.0) showing (a) cyclic voltammograms recorded at scan rates from 10 to 150 mV s^–1^, (b) plots of *I*_pa_ and *I*_pc_ versus scan rate *v*, (c) plots of *I*_pa_ and *I*_pc_ versus *v* ^1/2^, and (d) plot of *E*_p_ versus log_10_ *v*.

To investigate mass transport at the analyte–sensor interface, the Randles-Ševčík equation was applied. When the peak current varies linearly with the square root of the scan rate, the process is diffusion-controlled. Conversely, a direct linear dependence between the peak current and the scan rate suggests adsorption control.^58,65^ In Figure 5b,c, the linear relationships between the scan rate and the square root of the scan rate, plotted against the peak currents, yielded determination coefficients (*R* ^2^) close to each other, with values of 0.9876 and 0.9909 for the scan rate, and 0.9917 and 0.9889 for the square root of the scan rate. These results indicate a mixed mechanism involving both diffusion and adsorption.^58,65^

To confirm these findings, the relationship between the logarithm of the peak current (log *I*_pa_ and log *I*_pc_) and the logarithm of the scan rate (log_10_ *v*) was analyzed, as illustrated in Figure S7. In this study, the slopes obtained were 0.8123 (oxidation) and 0.7858 (reduction), indicating a mixed control with contributions from both diffusion and adsorption involving DA and the GCE/Nafion/NiNPs electrochemical sensor.^58,65^

To determine the anodic and cathodic electron-transfer coefficients (α_a_ and α_c_), *E*_pa_ and *E*_pc_ were plotted against the logarithm of the scan rate (log_10_ *v*), as shown in Figure 5d. A linear correlation between these parameters was observed for scan rates up to 40 mV s^–1^. Based on the peak potential and applying Laviron’s theory for redox processes with adsorptive contributions, the following equations were used

1 $$o=\frac{2.303\mathrm{RT}}{\left(1-\alpha \right)\mathrm{nF}}$$

2 $$p=\frac{2.303\mathrm{RT}}{\alpha \mathrm{nF}}$$

where (*o*) and (*p*) are the slopes, (*n*) is the number of electrons transferred, (*R*) is the universal gas constant (8.314 J mol^–1^ K^–1^), (*T*) is the temperature (298 K), (*F*) is the Faraday constant (96,485 C mol^–1^), and (α) is the electron-transfer coefficient. For an ideal irreversible process, the value of (α) is often assumed to be 0.5.^58^ Using this value as a reference and applying eqs 1 and 2, the number of electrons involved in the oxidation and reduction of DA at the GCE/Nafion/NiNPs sensor was estimated. The calculated values were 1.02 for oxidation and 0.91 for reduction, which can be approximated to 1.

Based on the above information, the experimental electron-transfer coefficients (α_a_ and α_c_) were calculated. By rewriting eqs 1 and 2, the experimental electron-transfer coefficients were determined as 0.49 for oxidation and 0.46 for reduction. These values are close to the theoretical value of 0.5, indicating that the system exhibits characteristics of a *quasi*-reversible process.^58,65^

Although these results suggest that the redox process of DA involves an apparent one-electron/one-proton rate-determining step under the selected experimental conditions, the mechanistic proposal requires further consideration. The applied potential window plays a crucial role in defining the nature of the electrochemical transformations that are observed. Within the potential window explored here, the redox behavior can be attributed predominantly to the DA/quinone couple.^66,67^ However, it is well-established that if the potential window is expanded toward more negative potentials, secondary processes such as the polymerization of DA may occur, leading to the formation of polydopamine films on the electrode surface.^67,68^ This phenomenon not only modifies the electrode/electrolyte interface but also introduces additional adsorption contributions, potentially altering the kinetics and mechanisms of the electron-transfer process.

Therefore, the electrochemical response of DA at the GCE/Nafion/NiNPs sensor is governed by a mixed regime involving both diffusion and adsorption. The apparent 1e^–^/1H^+^ ratio obtained from Laviron analysis should not be interpreted as the overall reaction stoichiometry, but rather as a kinetic feature of the rate-determining step under the experimental conditions. The global oxidation of DA remains a 2e^–^/2H^+^ process. Accordingly, the mechanism proposed in Figure 6 is consistent with previous reports.^24,67,69^

**Figure 6:**
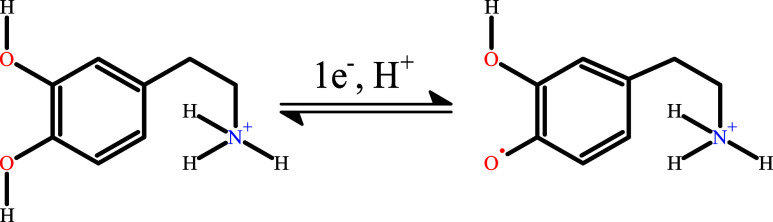
Proposed rate-determining step for dopamine oxidation at the GCE/Nafion/NiNPs sensor.

A more detailed analysis of the phase composition indicates that the NiNPs should be regarded as a hydroxide-dominated multiphase system rather than a simple metallic core–shell structure. XRD results supported by Rietveld refinement, together with XPS analysis, demonstrate that Ni­(OH)_2_ is the predominant phase (Ni:Ni­(OH)_2_:NiO ≈ 3.3:93.4:3.3), while metallic Ni and NiO are present only as minor components. This compositional distribution suggests that the electrochemical response is primarily governed by hydroxide/oxyhydroxide surface chemistry, with metallic and oxide phases playing secondary roles within the system. Taken together, these features establish a multiphase framework that underpins the overall electrochemical behavior.

From a mechanistic standpoint, the enhanced sensing performance of the GCE/Nafion/NiNPs electrode can be attributed to a synergistic interplay between electrostatic, adsorption, and electrocatalytic effects. The negatively charged Nafion matrix promotes the preconcentration of protonated DA at the electrode interface, increasing its local availability. Simultaneously, surface analysis (XPS and XRD) indicates that the NiNPs are predominantly composed of Ni­(OH)_2_/NiOOH species, which provide redox-active Ni­(II)/Ni­(III) sites. These redox couples are well-known for their electrochemical activity and have been widely associated with catalytic oxidation processes in nickel-based systems.^52–54^ In particular, Ni­(OH)_2_/NiOOH phases have been reported as effective electrocatalytic centers for the oxidation of catechol-containing molecules, including DA, through surface-mediated pathways.^28,59,60^ In this context, the metallic Ni domains may contribute to improved electrical conductivity and charge transport within the composite, while the oxidized phases govern the interfacial redox process, a synergistic behavior commonly observed in multiphase nickel-based electrocatalysts.^52,53^ This combined effect explains the observed quasi-reversible behavior and supports the mixed adsorption–diffusion mechanism identified from scan rate analysis.

### 3.4 Electrochemical Method Development and Preliminary Studies in Synthetic Urine

Based on previous studies of scan rate, a value of 50 mV s^–1^ was chosen for DA determination due to the best compromise between peak current and voltammogram resolution, which significantly contributes to the selectivity of the analytical method. Thus, the determination of DA was performed by CV with the addition of the analyte to the electrochemical cell, and the corresponding voltammograms were generated at increasing proportional analyte concentrations. Figure 7a,b show the voltammograms for DA over a range of 0.25 to 100 μmol L^–1^ and 150 to 350 μmol L^–1^, respectively. The graphs were displayed separately for better visual identification of the voltammograms, which were used to construct the calibration curve (Figure 7c).

**Figure 7:**
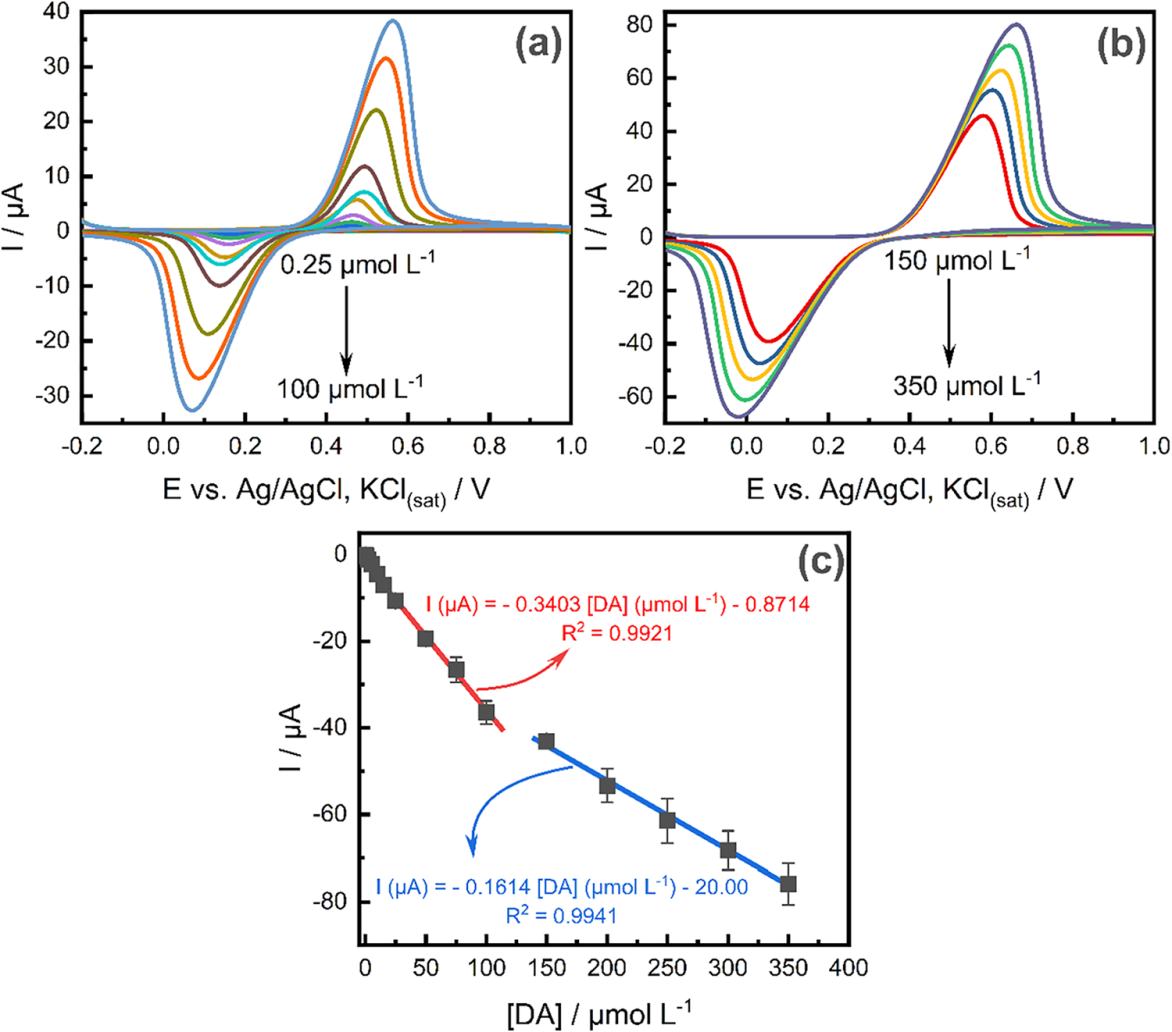
Calibration curve of DA for GCE/Nafion/NiNPs. (a) CV measurements at varying DA concentrations (0.25 to 100 μmol L^–1^), (b) CV measurements at higher DA concentrations (150 to 350 μmol L^–1^), and (c) correlation between Ipc and the concentration of DA for the two ranges (*n* = 3). Experimental conditions: B–R buffer 0.1 mol L^–1^ (pH 3.0). Scan rate: 50 mV s^–1^.

The calibration curve for *I*_pc_ as a function of DA concentration ([DA]) exhibited two linear regions (*n* = 3):

From 0.25 to 100 μmol L^–1^: I (μA) = −0.3403­[DA] – 0.8714 (*R* ^2^ = 0.9921)

From 150 to 350 μmol L^–1^: I (μA) = −0.1614­[DA] – 20.00 (*R* ^2^ = 0.9941)

The presence of two distinct linear regions is frequently reported in electrochemical sensing systems and is generally associated with changes in interfacial processes as the analyte concentration increases.^70–72^ At low DA concentrations, the enhanced sensitivity can be attributed to efficient interfacial accumulation promoted by the negatively charged Nafion matrix, which favors the electrostatic preconcentration of protonated DA (DA^+^) near the electrode surface, combined with the high density of active sites provided by the NiNPs. Under these conditions, the electrode surface operates far from saturation, resulting in a steeper calibration slope.

As the DA concentration increases, the progressive occupation of active sites and the possible adsorption of oxidation products lead to partial surface blocking, reducing the availability of active sites, as reported in previous studies.^72^ Consequently, the contribution of mass transport from the bulk solution becomes more relevant, leading to a decrease in sensitivity and the appearance of a second linear region with a lower slope. Similar dual linear responses have been widely reported in the literature and are commonly attributed to surface coverage effects, competitive adsorption, and changes in mass transport contributions at higher concentrations.^70,71^ In the present system, this behavior is consistent with the mixed adsorption–diffusion mechanism discussed previously and is further reinforced by the combined effects of Nafion-mediated preconcentration and Ni­(OH)_2_/NiOOH electrocatalytic sites.

The calculated LOD and LOQ were, respectively, 0.092 and 0.28 μmol L^–1^. The cathodic peak was used as the analytical signal to minimize possible contributions from electroactive interferents whose oxidation potentials may overlap with the anodic response of DA.

Table 1 presents sensors modified with different nickel materials that were used for DA determination. To our knowledge, the GCE/Nafion/NiNPs sensor is the first reported in the literature using NiNPs synthesized by pulsed laser ablation as the sensing modifier agent for DA detection.

**Table 1:** Developed Sensors Applied to DA Determination^a^

Sensor	Technique	Linear range (μmol L^–1^)	LOD (nmol L^–1^)	Sample	refs
Ni/C/GCE	DPV	1–55	50	Pharmaceuticals and fetal bovine serum	^73^
NLF/ITO	AMP	0.5–5	8	Dopaminergic cells	^74^
NiFeP	SWV	0.01–1	0.3	Pharmaceuticals	^75^
GCE/EG–Ni-Au (NPs)	SWV	0.2–100	100	None	^76^
NiO/CoO@PCNs/CNTs/erGO/GCE	DPV	0.1–22.0	45	Human blood serum	^77^
Ni@CNF/SPCE	CV	0.1–10	110	Pharmaceutical, human blood serum, and urine	^78^
	DPV	0.1–10	30		
Au@NiS_2_–FTO	AMP	0.1–1000	1	None	^79^
GCE/Nafion/NiNPs	CV	0.25–100	92	Synthetic human urine	This work

a DPV: differential pulse voltammetry; SWV: square wave voltammetry; AMP: amperometry; Ni/C/GCE: glassy carbon electrode modified with carbon-supported Ni nanoparticles; NLF/ITO: indium tin oxide glass modified with NiO-lacy flower-like structure; NiFeP: NiFe phosphides/phosphates-based flexible electrochemical sensor; GCE/EG–Ni-Au­(NPs): glassy carbon electrode modified with graphene oxide and nickel nanoparticles generated by cyclic voltammetry with posterior loading with gold nanoparticles via galvanic replacement; NiO/CoO@PCNs/CNTs/erGO/GCE: glassy carbon electrode modified with 3D flake nickel oxide/cobalt oxide@porous carbon nanosheets/carbon nanotubes/electrochemically reduced graphene oxide composites; Ni@CNF/SPCE: screen-printed carbon electrode modified with carbon nanofiber-supported nickel nanoparticles; Au@NiS2-FTO: fluorine-doped tin oxide glass modified with a hydrothermal method for synthesizing nickel disulfide followed by the deposition of gold nanoparticles by physical vapor deposition.

A closer examination of the reported systems reveals that most nickel-based sensors rely on chemically synthesized nanostructures combined with conductive supports (e.g., graphene, carbon nanofibers, carbon nanotubes) or secondary metallic components (e.g., Au), typically requiring multistep fabrication processes to enhance electrocatalytic performance. In contrast, the sensor proposed in this work employs NiNPs produced by PLAL, a method that generates ligand-free NPs with clean and highly reactive surfaces while preserving the intrinsic Ni/NiO/Ni­(OH)_2_ multiphase structure. This eliminates the need for additional functionalization or a complex composite formation. Despite this simplified and greener synthesis route, the sensor exhibits a comparable linear range and an LOD, while moderate relative to some advanced composite systems, reflecting the simplicity of the single-material, ligand-free fabrication approach. Therefore, the results indicate that the intrinsic surface properties of PLAL-derived NiNPs can effectively compensate for the absence of auxiliary conductive or catalytic materials, representing a straightforward and efficient strategy for DA electrochemical sensing.

For analytical evaluation, DA sensing was evaluated using CV and the proposed GCE/Nafion/NiNPs electrochemical sensor under optimized conditions. The quantification of DA was carried out in triplicate for three levels of concentration in spiked synthetic urine samples, namely 1.0, 2.0, and 3.0 μmol L^–1^ under the same experimental conditions used for the calibration curves (Figure S8). The results in Table 2 show that the recovery percentage ranges between 93.5 and 104.4%, in line with the Horwitz trumpet diagram.^80^ Thus, the developed electrochemical method for DA determination using the GCE/Nafion/NiNPs sensor shows good accuracy for synthetic urine sample analyses.

**Table 2:** Recovery Rates of DA in Synthetic Urine Samples (1:50 Dilution) Applying the GCE/Nafion/NiNPs Sensor (*n* = 3)

Sample	Theoretical (μmol L^–1^)	Experimental (μmol L^–1^)	Recovery (%)^a^	RSD (%)
1	1.0	0.99	99.0	±0.7
2	2.0	1.87	93.5	±3.9
3	3.0	3.13	104.4	±3.4

a Recovery = (Experimental value/Theoretical value) × 100.

The method exhibited good precision, as evidenced by the relative standard deviation (RSD) values ranging from 0.7% to 3.9%, indicating acceptable reproducibility across the tested concentrations. Notably, the physiological concentration range of DA in human urine typically falls between 0.3 and 3.1 μmol L^–1^.^81^ This suggests that the developed sensor remains effective in real-world scenarios where sample dilution may be required to minimize matrix interferences without compromising detection capability.

### 3.5 Repeatability and Selectivity Studies

The repeatability of the electrochemical sensor was evaluated through inter- and intraday experiments. Interday and intraday repeatability were evaluated using five replicate measurements (*n* = 5) on different days each and the same day, respectively. The relative standard deviation (RSD) of the current peak monitoring for interday analysis was 8.2, 8.4, and 8.5% for [DA] of 1.0, 5.0, and 10.0 μmol L^–1^, respectively (Figure S9a). During the intraday investigation, the RSD values achieved were 9.4, 7.5, and 4.7% for DA concentrations of 1.0, 5.0, and 10 μmol L^–1^, in that order (Figure S9b). Thus, both repeatability experiments showed precise RSD values for the developed electrochemical method.^80,82^

Some potential interferents that can be found in urine samples were tested in the presence of 0.5 μmol L^–1^ of DA in a synthetic urine sample.^83^ For these experiments, the concentration of each compound was set at 10 times higher than that of DA. Cyclic voltammograms were acquired in the presence of histamine, creatinine, L-tryptophan, glucose, ascorbic acid, MgCl_2_, and CaCl_2_ (Figure S10a–g). The relative current responses of *I*_pc_ showed changes lower than 15% relative to the DA response for all substances (Figure S10h), indicating that the use of the GCE/Nafion/NiNPs sensor in the developed electrochemical method is selective for DA determination in synthetic human urine.^80,82^ These results reinforce its promising features for analyses in synthetic biological matrices.

While these results confirm the selectivity and applicability of the GCE/Nafion/NiNPs sensor in complex biological matrices, some challenges remain for NiNP-based electrochemical sensing platforms. In particular, long-term operational stability, precise control over surface oxidation states, and reproducibility of the active Ni/NiO/Ni­(OH)_2_ interfaces may limit their broader implementation. Future studies should therefore focus on tuning nanoparticle surface chemistry through controlled PLAL parameters, postsynthesis treatments, or hybrid composite architectures to further enhance durability, selectivity, and suitability for advanced sensing applications.

## 4 Conclusions

This study presents the synthesis of ligand-free NiNPs via PLAL and their application in the electrochemical detection of DA. The resulting NiNPs, stabilized in ultrapure water without surfactants, were integrated into a GCE/Nafion platform, forming a composite with enhanced electrochemical performance. Electrochemical analysis revealed that Nafion imparted a favorable surface charge to the NiNPs, promoting strong electrostatic interactions with DA and enabling a mixed adsorption–diffusion mechanism. The method based on CV and the GCE/Nafion/NiNPs electrochemical sensor exhibited high sensitivity (LOD: 92 nmol L^–1^; linear range: 0.25–100 μmol L^–1^), satisfactory selectivity (interference <15%), and reproducibility (RSD ≤ 10%) in synthetic urine. This work introduces a sustainable and ligand-free approach for producing electrochemically active NiNPs and demonstrates their potential as a green and robust sensing platform for neurotransmitter detection in complex matrices.

## Supplementary Material



## Data Availability

The authors confirm that the data supporting the findings of this study are included within the article and its Supporting Information. Furthermore, raw data that underpin the results of this study are available from the corresponding author upon reasonable request.

## Notes

Rietveld refinement; full Raman spectrum; XPS survey spectrum and Ni 2p/O 1s fitting parameters; DLS size distribution and zeta-potential data; pH, supporting electrolyte, buffer-concentration, and scan-rate studies; synthetic urine recovery voltammograms; repeatability and interference studies (PDF)

## References

[1] Moya C., Ara J., Labarta A., Batlle X. (2024). Unraveling the Magnetic Properties
of NiO Nanoparticles: From Synthesis to Nanostructure. Magnetism.

[2] Supin K. K., Namboothiri P., Vasundhara M. (2024). Complex Magnetic
Behaviour and Photocatalytic
Response in Narrowed Band Gap NiO Nanoparticles Synthesized by Novel
Lepidagathis Ananthapuramensis Leaf Extract. Mater. Sci. Eng., B.

[3] Xi Z., Wei K., Wang Q., Kim M. J., Sun S., Fung V., Xia X. (2021). Nickel–platinum Nanoparticles
as Peroxidase Mimics with a
Record High Catalytic Efficiency. J. Am. Chem.
Soc..

[4] Chen Y., Mu W., Meng J., Huang Y., Bi X., Yang R., Lei X., Luo S. (2024). Nickel-Copper Bimetallic Oxide Nanoparticles Prepared
by Simple Coprecipitation Method as High Performance Electrode Materials
for Asymmetric Supercapacitors. Langmuir.

[5] Guerrero-Ortega L. P. A., Ramírez-Meneses E., Betancourt I., Lartundo-Rojas L., Mendoza-Cruz R., Torres-Huerta A. M., Domínguez-Crespo M. A. (2023). Effect of Alkyl Chain Length of Amines
on the Micro-Structural and Magnetic Properties of Stabilized Ni-NiO
Nanoparticles. J. Inorg. Organomet. Polym. Mater..

[6] Liu S., Tam S. K., Ng K. M. (2021). Dual-Reductant Synthesis of Nickel
Nanoparticles for Use in Screen-Printing Conductive Paste. J. Nanoparticle Res..

[7] Fujioka D., Ikeda S., Akamatsu K., Nawafune H., Kojima K. (2019). Preparation
of Ni Nanoparticles by Liquid-Phase Reduction to Fabricate Metal Nanoparticle–Polyimide
Composite Films. RSC Adv..

[8] Bouremana A., Mouaci S., Berriah A., Boutebina Z., Manseri A., Bensouilah A. (2022). High Yield
Solvothermal Synthesis
of Ni Nanoparticles: Structural, Microstructural, and Magnetic Properties. J. Nanoparticle Res..

[9] Saidi N. M., Khairudin A., Li L., Abdah M. A. A. M., Gerard O., Tan Y. S., Khalid M., Khan F., Mustafa M. N., Numan A. (2023). Performance
Comparison of 2D Nickel
Phosphate Nanoparticles Prepared via Sonochemical and Microwave-Assisted
Hydrothermal Routes for Supercapattery. J. Energy
Storage.

[10] Shaheen M. E., Abdelwahab A. Y. E. (2025). Laser Ablation in Liquids: A Versatile Technique for
Nanoparticle Generation. Opt. Laser Technol..

[11] Ricardo P. C., Vanoni C. R., Piotrowski M. J., Costa D. C., Padrão J., Zille A., Satulu V., Dinescu G., Carvalho Ó., Jost C. L., Silva F. S., Fredel M. C. (2026). Gold Nanoparticles
Synthesized by Laser Ablation in Saline Medium: An Assessment of Colloidal
Stability. Int. J. Precis. Eng. Manuf. Technol.
2026.

[12] Ricardo P. C., Vanoni C. R., Padrão J., Zille A., Satulu V., Mitu B., da Cunha
Pereira M. R., Jost C. L., Silva F. S., Fredel M. C. (2026). Engineering
Graphene Quantum Dots via Laser Ablation
for Epinephrine Sensing. Diamond Relat. Mater..

[13] De
Bortoli L. S., Vanoni C. R., Jost C. L., Mezalira D. Z., Fredel M. C. (2023). Stable and Ligand-Free Gold Nanoparticles Produced
by Laser Ablation as Efficient Electrocatalysts for Electrochemical
Sensing of Dopamine. J. Electroanal. Chem..

[14] Kongkaew S., Srilikhit A., Janduang S., Thipwimonmas Y., Kanatharana P., Thavarungkul P., Limbut W. (2024). Single Laser Synthesis
of Gold Nanoparticles-Polypyrrole-Chitosan on Laser-Induced Graphene
for Ascorbic Acid Detection. Talanta.

[15] Ricardo P. C., Nicolodelli G., Fredel M. C., Cavalcante P., Nicolodelli G., Ricardo P. C., Nicolodelli G., Fredel M. C. (2025). Tunable Photoluminescence
of Graphene Quantum Dots
Synthesized via Laser Ablation in Ethanol. J.
Lumin..

[16] Latif S., Jahangeer M., Razia D. M., Ashiq M., Ghaffar A., Akram M., El Allam A., Bouyahya A., Garipova L., Ali Shariati M., Thiruvengadam M., Azam Ansari M. (2021). Dopamine in
Parkinson’s Disease. Clin. Chim. Acta.

[17] Hou G., Hao M., Duan J., Han M. H. (2024). The Formation and Function of the
VTA Dopamine System. Int. J. Mol. Sci..

[18] Tai M. D. S., Gamiz-Arco G., Martinez A. (2024). Dopamine Synthesis and Transport:
Current and Novel Therapeutics for Parkinsonisms. Biochem. Soc. Trans..

[19] Howes O. D., Shatalina E. (2022). Integrating
the Neurodevelopmental and Dopamine Hypotheses
of Schizophrenia and the Role of Cortical Excitation-Inhibition Balance. Biol. Psychiatry.

[20] Wise R. A., Jordan C. J. (2021). Dopamine, Behavior,
and Addiction. J. Biomed. Sci..

[21] Alhusban A. A., Hammad A. M., Alzaghari L. F., Shallan A. I., Shnewer K. (2023). Rapid and
Sensitive HPLC–MS/MS Method for the Quantification of Dopamine,
GABA, Serotonin, Glutamine and Glutamate in Rat Brain Regions after
Exposure to Tobacco Cigarettes. Biomed. Chromatogr..

[22] Chen F., Fang B., Wang S. (2021). A Fast and
Validated HPLC Method
for Simultaneous Determination of Dopamine, Dobutamine, Phentolamine,
Furosemide, and Aminophylline in Infusion Samples and Injection Formulations. J. Anal. Methods Chem..

[23] Guiard B. P., Gotti G. (2024). The High-Precision Liquid Chromatography with Electrochemical Detection
(HPLC-ECD) for Monoamines Neurotransmitters and Their Metabolites:
A Review. Molecules.

[24] Lima A. R. S., Mikhraliieva A., Vanoni C. R., Nazarkovsky M., Xing Y., Couto M. T., Zaitsev V., Jost C. L. (2024). 2D-Network
of Boron-Functionalized N-Doped Graphene Quantum Dots for Electrochemical
Sensing of Dopamine. Diamond Relat. Mater..

[25] Sliesarenko V., Bren U., Lobnik A. (2024). Fluorescence Based Dopamine Detection. Sens. Actuators Rep..

[26] Choi Y., Jeon C. S., Kim K. B., Kim H. J., Pyun S. H., Park Y. M. (2023). Quantitative Detection of Dopamine
in Human Serum with
Surface-Enhanced Raman Scattering (SERS) of Constrained Vibrational
Mode. Talanta.

[27] Cheng T., Afshan N., Jiao J., Jiao J. (2024). Current Progress in
Aptamer-Based Sensors for the Detection of Protein Biomarkers in Neurodegenerative
Diseases. Biosens. Bioelectron. X.

[28] Roychoudhury A., Basu S., Jha S. K. (2016). Dopamine
Biosensor Based on Surface
Functionalized Nanostructured Nickel Oxide Platform. Biosens. Bioelectron..

[29] Ricardo P. C., Vanoni C. R., Lima A. R. S., Jost C. L., Padrão J., Silva F. S., Fredel M. C. (2025). Tailored Ligand-Free
Gold Nanoparticles:
Exploring Green Synthesis in Saline Media for the Electrochemical
Sensing of Serotonin. Electrochim. Acta.

[30] Long G. L., Winefordner J. D. (1983). Limit of Detection: A Closer Look at the IUPAC Definition. Anal. Chem..

[31] Liu L., Shou L., Yu H., Yao J. (2015). Mechanical Properties
and Corrosion Resistance of Vulcanized Silicone Rubber after Exposure
to Artificial Urine. J. Macromol. Sci. Part
B.

[32] Manyar S. S., Nangare S., Mahajan M. R., Khatik Z. K. A., Patil P. O. (2025). Cysteine-Mediated
N, S-Graphene Quantum Dots Adorned UiO-MOFs Nanocomposite: A Fast
and Ultrasensitive Fluorescence Sensor for Para-Amino Hippuric Acid. Luminescence.

[33] Arboleda D. M., Santillán J. M. J., Herrera L. J. M., Van
Raap M. B. F., Zélis P. M., Muraca D., Schinca D. C., Scaffardi L. B. (2015). Synthesis of Ni Nanoparticles by Femtosecond Laser
Ablation in Liquids: Structure and Sizing. J.
Phys. Chem. C.

[34] Lee S. J., Theerthagiri J., Choi M. Y. (2022). Time-Resolved Dynamics of Laser-Induced
Cavitation Bubbles during Production of Ni Nanoparticles via Pulsed
Laser Ablation in Different Solvents and Their Electrocatalytic Activity
for Determination of Toxic Nitroaromatics. Chem.
Eng. J..

[35] Mohammed S. R., Ali A. H. (2025). Using Laser Ablation To Prepare Nano Nickel And Studying
Optical Properties. J. Opt..

[36] Chowdhury, M. ; Singh, B. K. ; Vadnala, S. ; Tiwari, A. ; Tripathi, A. ; Rawat, R. Structural Evolution of Ni/NiO Nanostructures Synthesized by Pulsed Laser Ablation in Water Acad. Nano Sci. Mater. Technol. 2025; Vol. 2 4 10.20935/AcadNano7969.

[37] Aman A. W., Krishnan G., Sadiqi M. A., Alhajj M., Hidayat N. (2025). Facile Synthesis
of Copper, Nickel and Their Bimetallic Nanoparticles: Optical and
Structural Characterization. Discovery Nano.

[38] Sunny A., Balasubramanian K. (2021). Laser-Induced Phonon and Magnon Properties of NiO Nanoparticles:
A Raman Study. J. Raman Spectrosc..

[39] Sunny A., Balasubramanian K. (2020). Raman Spectral
Probe on Size-Dependent Surface Optical
Phonon Modes and Magnon Properties of NiO Nanoparticles. J. Phys. Chem. C.

[40] Biesinger M. C., Payne B. P., Lau L. W. M., Gerson A., Smart R. S. C. (2009). X-Ray
Photoelectron Spectroscopic Chemical State Quantification of Mixed
Nickel Metal, Oxide and Hydroxide Systems. Surf.
Interface Anal..

[41] Grosvenor A. P., Biesinger M. C., Smart R. S. C., McIntyre N. S. (2006). New Interpretations
of XPS Spectra of Nickel Metal and Oxides. Surf.
Sci..

[42] Nesbitt H. W., Legrand D., Bancroft G. M. (2000). Interpretation of
Ni2p XPS Spectra
of Ni Conductors and Ni Insulators. Phys. Chem.
Miner..

[43] Biesinger M. C., Lau L. W. M., Gerson A. R., Smart R. S. C. (2012). The Role of the
Auger Parameter in XPS Studies of Nickel Metal, Halides and Oxides. Phys. Chem. Chem. Phys..

[44] Peck M. A., Langell M. A. (2012). Comparison of Nanoscaled
and Bulk NiO Structural and
Environmental Characteristics by XRD, XAFS, and XPS. Chem. Mater..

[45] Chen L.-Y., Zhang L.-C., Zhou S., Rahman A., Guisbiers G. (2024). Synthesis
of Nickel-Based Nanoparticles by Pulsed Laser Ablation in Liquids:
Correlations between Laser Beam Power, Size Distribution and Cavitation
Bubble Lifetime. Metals.

[46] Mahfouz R., Aires F. J. C. S., Brenier A., Jacquier B., Bertolini J. C. (2008). Synthesis
and Physico-Chemical Characteristics of Nanosized Particles Produced
by Laser Ablation of a Nickel Target in Water. Appl. Surf. Sci..

[47] Filippov S. K., Khusnutdinov R., Murmiliuk A., Inam W., Zakharova L. Y., Zhang H., Khutoryanskiy V. V. (2023). Dynamic Light Scattering and Transmission
Electron Microscopy in Drug Delivery: A Roadmap for Correct Characterization
of Nanoparticles and Interpretation of Results. Mater. Horizons.

[48] Sizochenko N., Mikolajczyk A., Syzochenko M., Puzyn T., Leszczynski J. (2021). Zeta Potentials
(ζ) of Metal Oxide Nanoparticles: A Meta-Analysis of Experimental
Data and a Predictive Neural Networks Modeling. NanoImpact.

[49] Lorenzen A. L., Rossi T. S., Riegel-Vidotti I. C., Vidotti M. (2018). Influence of Cationic
and Anionic Micelles in the (Sono)­Chemical Synthesis of Stable Ni­(OH)­2
Nanoparticles: “In Situ” Zeta-Potential Measurements
and Electrochemical Properties. Appl. Surf.
Sci..

[50] Atta N. F., Galal A., El-Gohary A. R. M. (2021). An Innovative Design of Hydrazine
Hydrate Electrochemical Sensor Based on Decoration of Crown Ether/Nafion/Carbon
Nanotubes Composite with Gold Nanoparticles. J. Electroanal. Chem..

[51] Li Y., Pan F., Yin S., Tong C., Zhu R., Li G. (2022). Nafion Assisted
Preparation of Prussian Blue Nanoparticles and Its Application in
Electrochemical Analysis of L-Ascorbic Acid. Microchem. J..

[52] Deabate S., Henn F. (2005). Structural Modifications and Electrochemical
Behaviour of the β­(II)-Ni­(OH)­2/β­(III)-NiOOH
Redox Couple upon Galvanostatic Charging/Discharging Cycling. Electrochim. Acta.

[53] Sun W., Govindarajan N., Prajapati A., Huang J., Bemana H., Feaster J. T., Akhade S. A., Kornienko N., Hahn C. (2025). Insights into the Electrochemical
Oxidation and Reduction of Nickel
Oxide Surfaces. ACS Appl. Mater. Interfaces.

[54] Khateri M., Najafpour M. M. (2024). Oxygen-Evolution Reaction on Nickel Oxyhydroxide’s
Surface: Toward a Super Catalyst for Oxygen-Evolution Reaction with
Ultralow Overpotentials. ACS Appl. Energy Mater..

[55] Sunday C. E., Bilibana M., Qakala S., Tovide O., Molapo K. M., Fomo G., Ikpo C. O., Waryo T., Mbambisa G., Mpushe B., Williams A., Baker P. G. L., Vilakazi S., Tshikhudo R., Iwuoha E. I. (2014). Modulation of the Matrix Effect of
Nafion on Tris­(Bipyridine) Ruthenium­(II) Electrochemical Probes by
Functionalisation with 4-Nitrophenylazo Graphene-Gold Nanocomposite. Electrochim. Acta.

[56] Messias V. B., Modenez D. C. P., de
Souza Pereira C. E., Takeuchi R. M., dos Santos A. L. (2025). Practical
Considerations for Using Redox Probes in Electrochemical Sensor Characterization. Electrochim. Acta.

[57] Manasa G., Mahamiya V., Chakraborty B., Rout C. S. (2024). 2D/1D VSe2/MWCNT
Hybrid-Based Electrochemical Sensor for Carbendazim Quantification
of Environmental, Food, and Biological Samples. Microchim. Acta.

[58] Bard, A. J. ; Faulkner, L. R. ; White, H. S. Electrochemical Methods: Fundamentals and Applications, 3rd ed.; Wiley, 2022; p 1044.

[59] Nancy T. E. M., Kumary V. A. (2014). Synergistic Electrocatalytic Effect
of Graphene/Nickel
Hydroxide Composite for the Simultaneous Electrochemical Determination
of Ascorbic Acid, Dopamine and Uric Acid. Electrochim.
Acta.

[60] Zhang S., Fu Y., Sheng Q., Zheng J. (2017). Nickel–Cobalt Double Hydroxide
Nanosheets Wrapped Amorphous Ni­(OH)­2 Nanoboxes: Development of Dopamine
Sensor with Enhanced Electrochemical Properties. New J. Chem..

[61] Vanoni C. R., Goularte R. B., Lima A. R. S., Guedes N. B., Scheide M. R., Bazzo G. C., Parreira R. L. T., Caramori G. F., Nagurniak G. R., Dotto M. E. R., Stulzer H. K., Jost C. L. (2025). An Alumina-Modified
Glassy Carbon Electrode: A Robust Platform for Accurate Nimodipine
Detection in Pharmaceutical Applications. Anal.
Methods.

[62] Auinger M., Katsounaros I., Meier J. C., Klemm S. O., Biedermann P. U., Topalov A. A., Rohwerder M., Mayrhofer K. J. J. (2011). Near-Surface
Ion Distribution and Buffer Effects during Electrochemical Reactions. Phys. Chem. Chem. Phys..

[63] de
Macedo J. F., Alves A. A. C., Sant’Anna M. V. S., Cunha F. G. C., Oliveira G., de A. R., Lião L. M., Sussuchi E. M. (2022). Electrochemical Determination of Carbendazim in Grapes
and Their Derivatives by an Ionic Liquid-Modified Carbon Paste Electrode. J. Appl. Electrochem..

[64] Nurzulaikha R., Lim H. N., Harrison I., Lim S. S., Pandikumar A., Huang N. M., Lim S. P., Thien G. S. H., Yusoff N., Ibrahim I. (2015). Graphene/SnO2 Nanocomposite-Modified
Electrode for
Electrochemical Detection of Dopamine. Sens.
Bio-Sensing Res..

[65] Brett, C. M. A. ; Brett, A. N. O. Principles, Methods, and Applications. In Electrochemistry; Oxford University Press, 1993; Vol. 67, p 444.

[66] Medintz I. L., Stewart M. H., Trammell S. A., Susumu K., Delehanty J. B., Mei B. C., Melinger J. S., Blanco-Canosa J. B., Dawson P. E., Mattoussi H. (2010). Quantum-Dot/Dopamine Bioconjugates
Function as Redox Coupled Assemblies for in Vitro and Intracellular
PH Sensing. Nat. Mater. 2010 98.

[67] Olejnik A., Ficek M., Siuzdak K., Bogdanowicz R. (2022). Multi-Pathway
Mechanism of Polydopamine Film Formation at Vertically Aligned Diamondised
Boron-Doped Carbon Nanowalls. Electrochim. Acta.

[68] Jaramillo A. M., Barrera-Gutiérrez R., Cortés M. T. (2020). Synthesis,
Follow-Up, and Characterization of Polydopamine-like Coatings Departing
from Micromolar Dopamine- o-Quinone Precursor Concentrations. ACS Omega.

[69] Bacil R. P., Chen L., Serrano S. H. P., Compton R. G. (2020). Dopamine Oxidation
at Gold Electrodes: Mechanism and Kinetics near Neutral PH. Phys. Chem. Chem. Phys..

[70] Su C. H., Sun C. L., Liao Y. C. (2017). Printed
Combinatorial Sensors for
Simultaneous Detection of Ascorbic Acid, Uric Acid, Dopamine, and
Nitrite. ACS Omega.

[71] Chantaramethakul J., Janbooranapinij K., Sungprasit C., Jongprateep O., Niamlang S., Manuspiya H., Panomsuwan G. (2025). Electrochemical
Detection of Nitrite Ions Using Gold Nanoparticles Supported on Multiwalled
Carbon Nanotube/Reduced Graphene Oxide Composites. ACS Appl. Nano Mater..

[72] Lisnund S., Blay V., Chansaenpak K., Monkrathok J., Pinyou P. (2025). Simultaneous Electrochemical Determination
of Dopamine,
Acetaminophen, and Caffeine with a PVP/RGO-Modified Electrode. ACS Omega.

[73] He W., Ding Y., Zhang W., Ji L., Zhang X., Yang F. (2016). A Highly Sensitive Sensor for Simultaneous Determination of Ascorbic
Acid, Dopamine and Uric Acid Based on Ultra-Small Ni Nanoparticles. J. Electroanal. Chem..

[74] Emran M. Y., Shenashen M. A., Mekawy M., Azzam A. M., Akhtar N., Gomaa H., Selim M. M., Faheem A., El-Safty S. A. (2018). Ultrasensitive
In-Vitro Monitoring of Monoamine Neurotransmitters from Dopaminergic
Cells. Sens. Actuators, B.

[75] Thakur N., Chaturvedi A., Mandal D., Nagaiah T. C., Li R., Chemcomm, Communication C. (2020). Ultrasensitive
and Highly Selective Detection of Dopamine by a NiFeP Based Flexible
Electrochemical Sensor. Chem. Commun..

[76] Hassine C. B. A., Kahri H., Barhoumi H. (2020). Enhancing
Dopamine Detection Using
Glassy Carbon Electrode Modified with Graphene Oxide, Nickel and Gold
Nanoparticles. J. Electrochem. Soc..

[77] Zhang L., Tang J., Li J., Li Y., Yang P., Zhao P., Fei J., Xie Y. (2023). A Novel Dopamine
Electrochemical
Sensor Based on 3D Flake Nickel Oxide/ Cobalt Oxide @ Porous Carbon
Nanosheets/Carbon Nanotubes/Electrochemical Reduced of Graphene Oxide
Composites Modified Glassy Carbon Electrode. Colloids Surf., A.

[78] Sharma V., Singh P., Kumar A., Gupta N. (2023). Electrochemical Detection
of Dopamine by Using Nickel Supported Carbon Nanofibers Modified Screen
Printed Electrode. Diamond Relat. Mater..

[79] Ma C., Wen Y., Qiao Y., Shen K. Z., Yuan H. (2024). A Dopamine Detection
Sensor Based on Au-Decorated NiS2 and Its Medical Application. Molecules.

[80] Mermet, J.-M. ; Otto, M. ; Valcárcel Cases, M. Analytical Chemistry : A Modern Approach to Analytical. In (2004). Analytical Chemistry : A Modern Approach to Analytical Science; SMermet, J.-M. ; Otto, M. ; Valcárcel Cases, M. , Eds.; Wiley-VCH, 2004; p 1181.

[81] Steckl A. J., Ray P. (2018). Stress Biomarkers in Biological Fluids and Their Point-of-Use Detection. ACS Sens..

[82] Latimer, G. W. Guidelines for Standard Method Performance Requirements. In Official Methods of Analysis of AOAC INTERNATIONAL; Oxford University Press, 2023; pp AF-1–AF-18 10.1093/9780197610145.005.006.

[83] Beatto T. G., Gomes W. E., Etchegaray A., Gupta R., Mendes R. K. (2023). Dopamine
Levels Determined in Synthetic Urine Using an Electrochemical Tyrosinase
Biosensor Based on ZnO@Au Core–Shell. RSC Adv..

