# Supplementary material for: Nickel-Based Nanoparticles Synthesized by Pulsed Laser Ablation in Liquid with Multiphase Structure for Electrochemical Dopamine Sensing
Source: ACS Omega. 2026 Jun 20;11(26):39429–44. doi: 10.1021/acsomega.6c05305 (PMC13347396; doi:10.1021/acsomega.6c05305)
Supplement: Supplementary file 1 [file ao6c05305_si_001.pdf]

Supporting Information

Nickel-Based Nanoparticles Synthesized by Pulsed Laser Ablation in Liquid with Multiphase Structure for Electrochemical Dopamine Sensing

Tomas Raphael Woida, Philipi Cavalcante Ricardo, Caio Raphael Vanoni, Adriano Rogério Silva Lima, Kurosch Rezwan, Cristiane Luisa Jost, Márcio Celso Fredel.

* Corresponding author:

e-mail address: m.fredel@ufsc.br

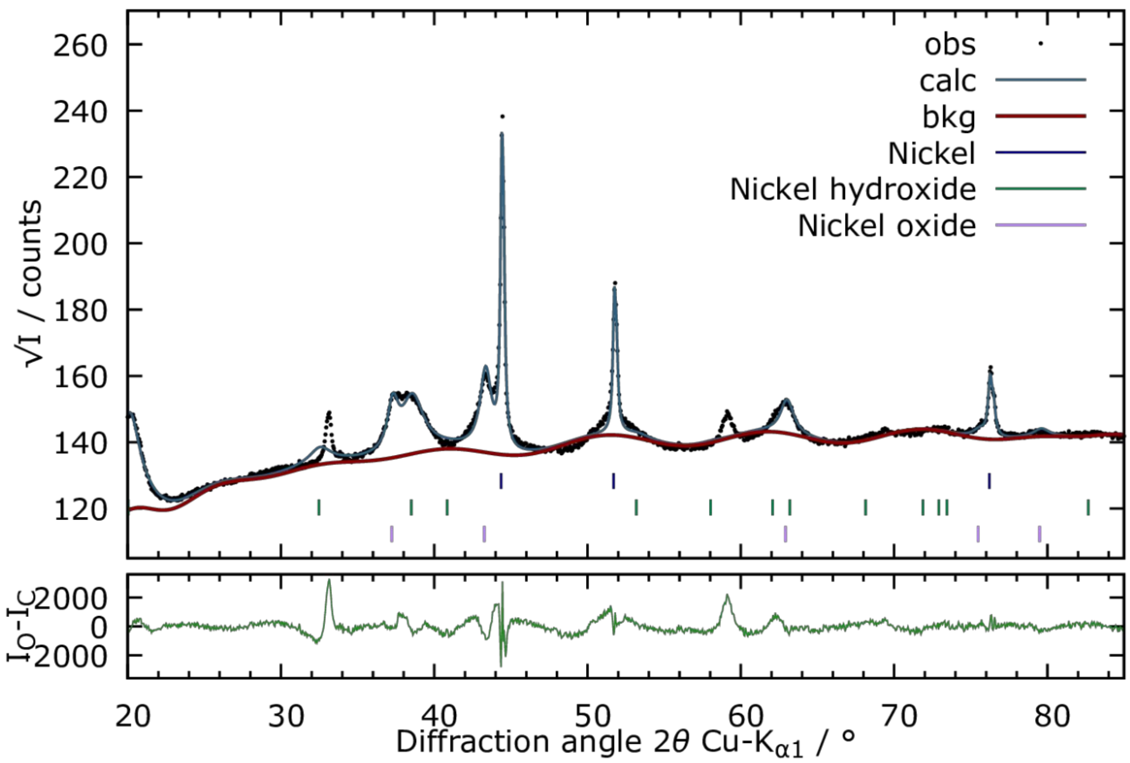

Figure S1. Rietveld refinement of the XRD pattern of the oven-dried powder. Experimental data (light blue circles), calculated profile (red line), and difference curve (green line) are shown. Bragg positions are indicated as tick marks for Ni (fcc, dark blue), NiO (green), and $Ni(OH)_2$ (purple).

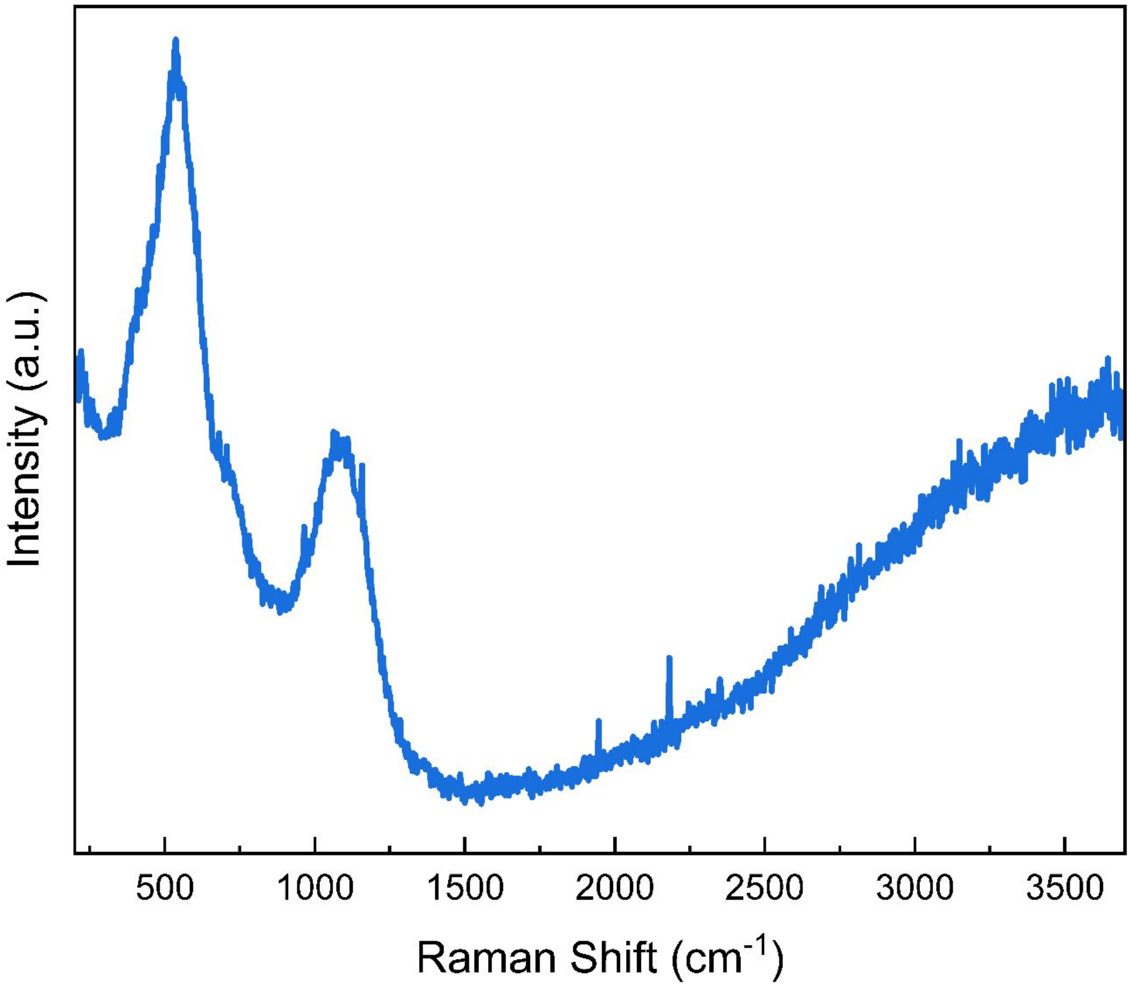

Figure S2. Raman spectrum of the oven-dried NiNPs. The spectrum is dominated by a broad LO mode of NiO (~561.7 cm^{-1}), with additional contributions from TO, SO, 2TO, and 2LO modes. The pronounced band broadening indicates structural disorder and phonon confinement in nanocrystalline NiO.

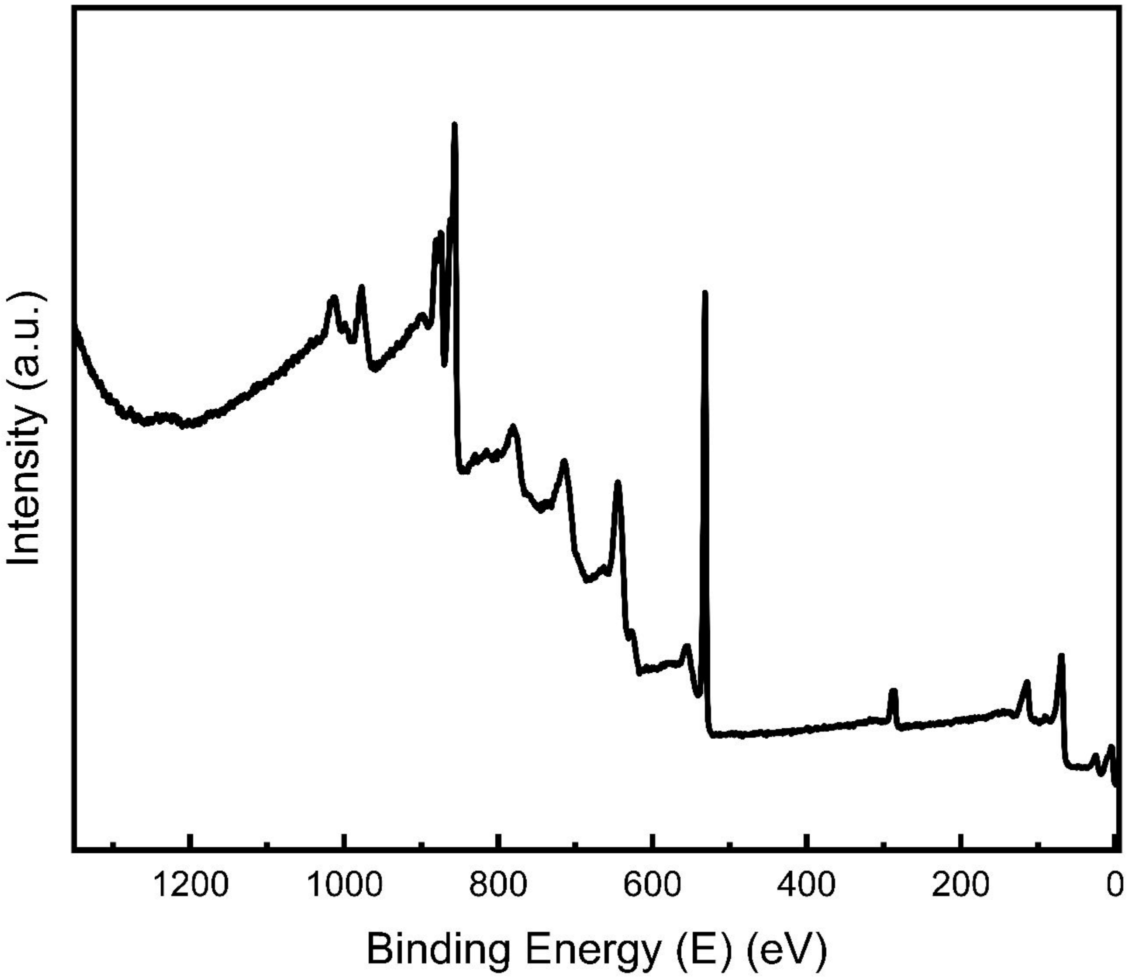

Figure S3. XPS survey spectrum of the oven-dried NiNPs, confirming Ni and O as the main surface elements.

Table S1. Peak fitting parameters obtained from the deconvolution of the Ni 2p XPS spectrum of NiNPs synthesized by PLAL. Binding energies, full width at half maximum (FWHM), and relative peak areas are reported for each component. The assignments are based on literature values for NiO, $Ni(OH)_2$, and NiOOH species, as well as shake-up satellite features.

**Component**	**Assignment**	**Binding Energy (eV)**	**FWHM (eV)**	**Relative Area (%)**
Ni $2p_{3/2}$ (B1)	Ni^{2+} (NiO / partially reduced Ni)	853.30	2.00	1.44
Ni $2p_{3/2}$ (B2)	Ni^{2+} (NiO-like)	855.04	2.18	7.72
Ni $2p_{3/2}$ (A1)	Ni^{2+} ($Ni(OH)_2$)	856.02	1.96	10.93
Ni $2p_{3/2}$ (A2)	Ni^{3+} (NiOOH)	857.20	2.50	13.20
Ni $2p_{3/2\ satellite}$	Shake-up	860.00	3.93	7.55
Ni $2p_{3/2\ satellite}$	Shake-up	861.85	2.87	5.87
Ni $2p_{3/2\ satellite}$	Shake-up	863.40	6.00	17.22
Ni $2p_{1/2}$	Ni^{2+}/Ni^{3+}	873.50	2.60	12.21
Ni $2p_{1/2\ satellite}$	Shake-up	876.00	2.58	3.35
Ni $2p_{1/2\ satellite}$	Shake-up	880.00	6.00	20.52

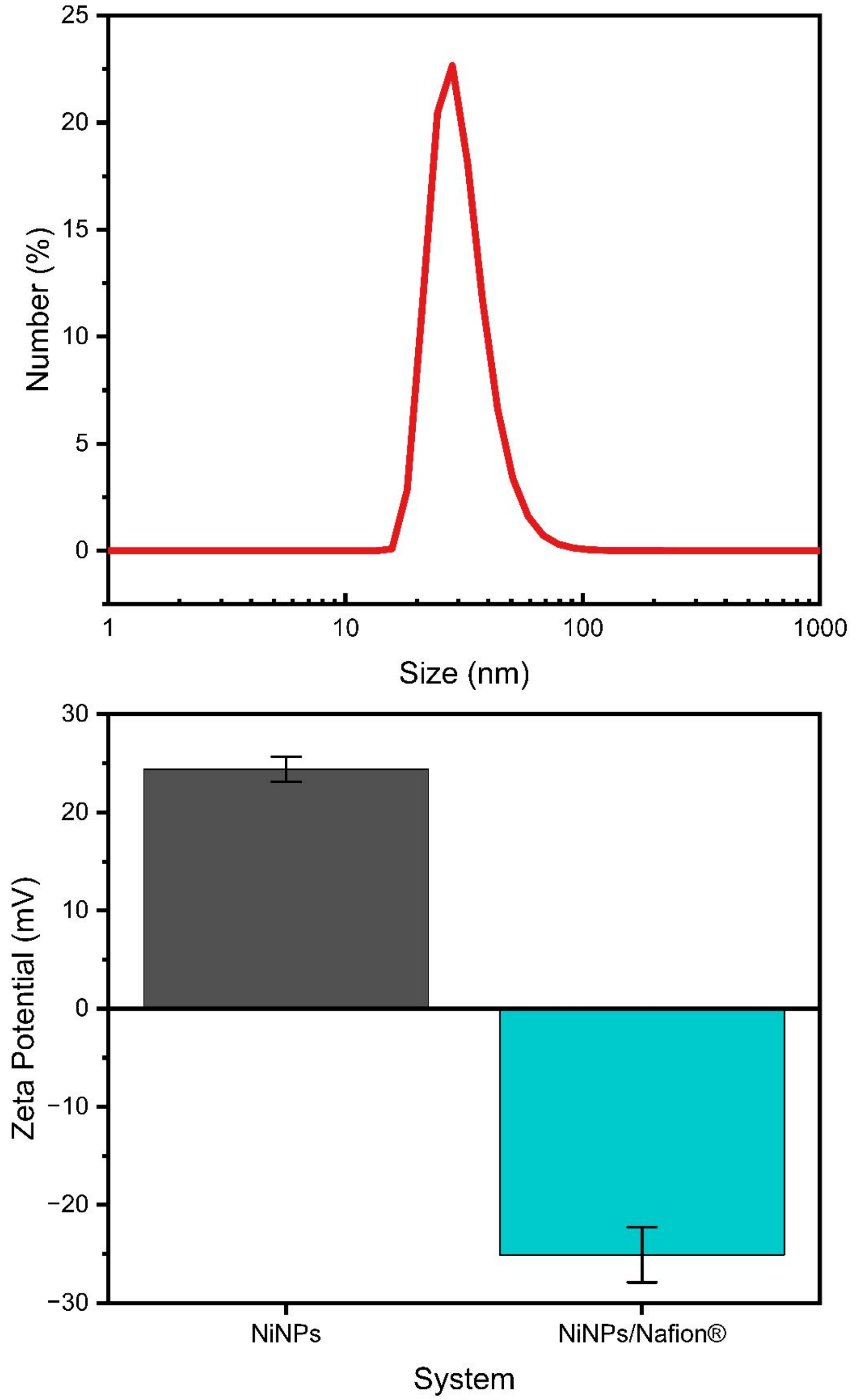

Figure S4. (a) DLS size distribution of the NiNPs, showing a narrow distribution centered at 26.9 ± 2.2 nm. (b) Zeta potential of as-prepared NiNPs (+24.4 mV) and Nafion/NiNPs (−25.13 mV), indicating a shift in surface charge upon incorporation into the polymer matrix.

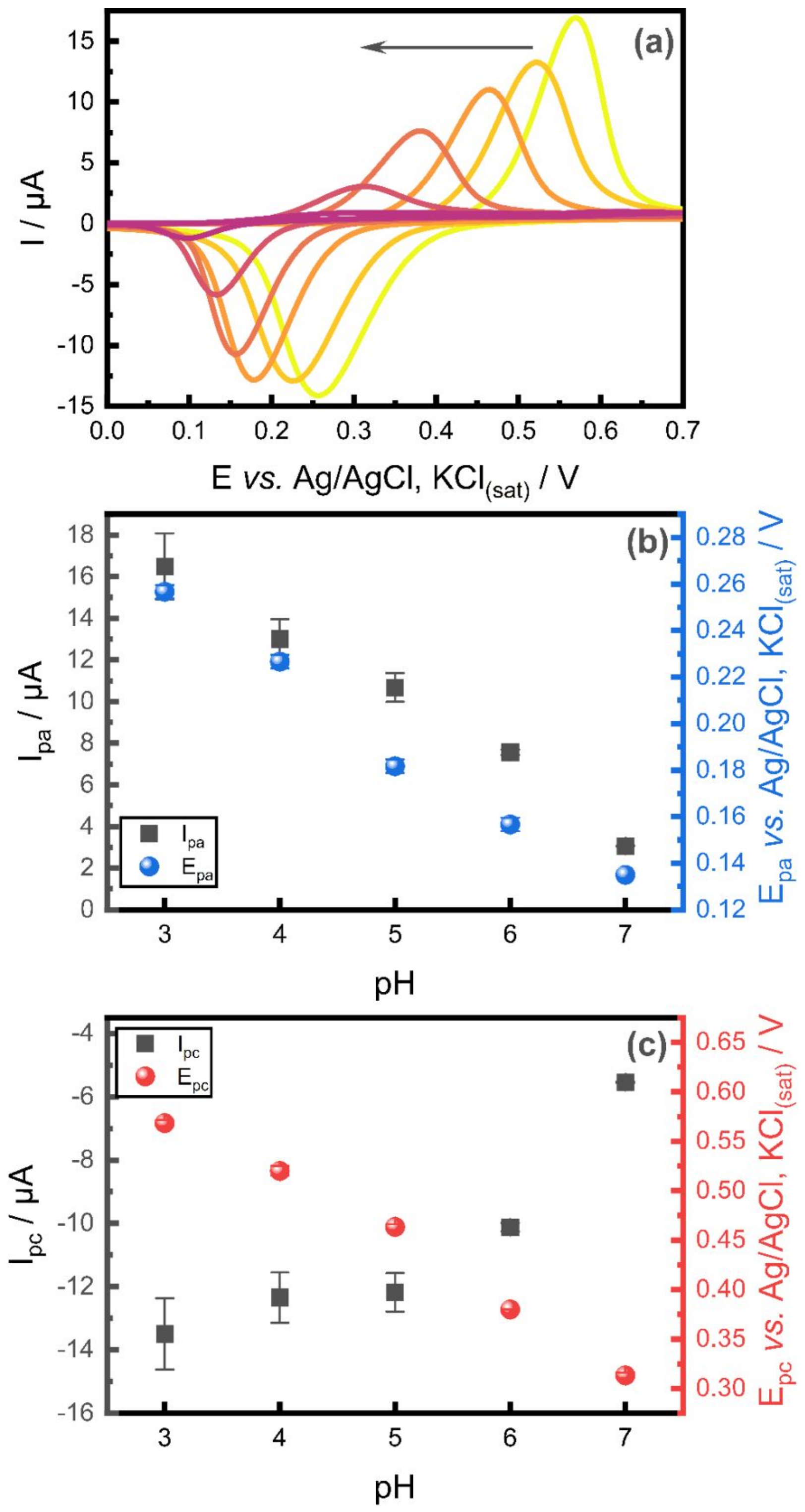

Figure S5. (a) CVs for 50 µmol L^{-1} DA at GCE/Nafion/NiNPs in B-R buffer at different pH values (3.0 - 7.0) and (b) variation of anodic peak potentials and currents as a function of pH, and (c) variation of cathodic peak potentials and currents as a function of pH.

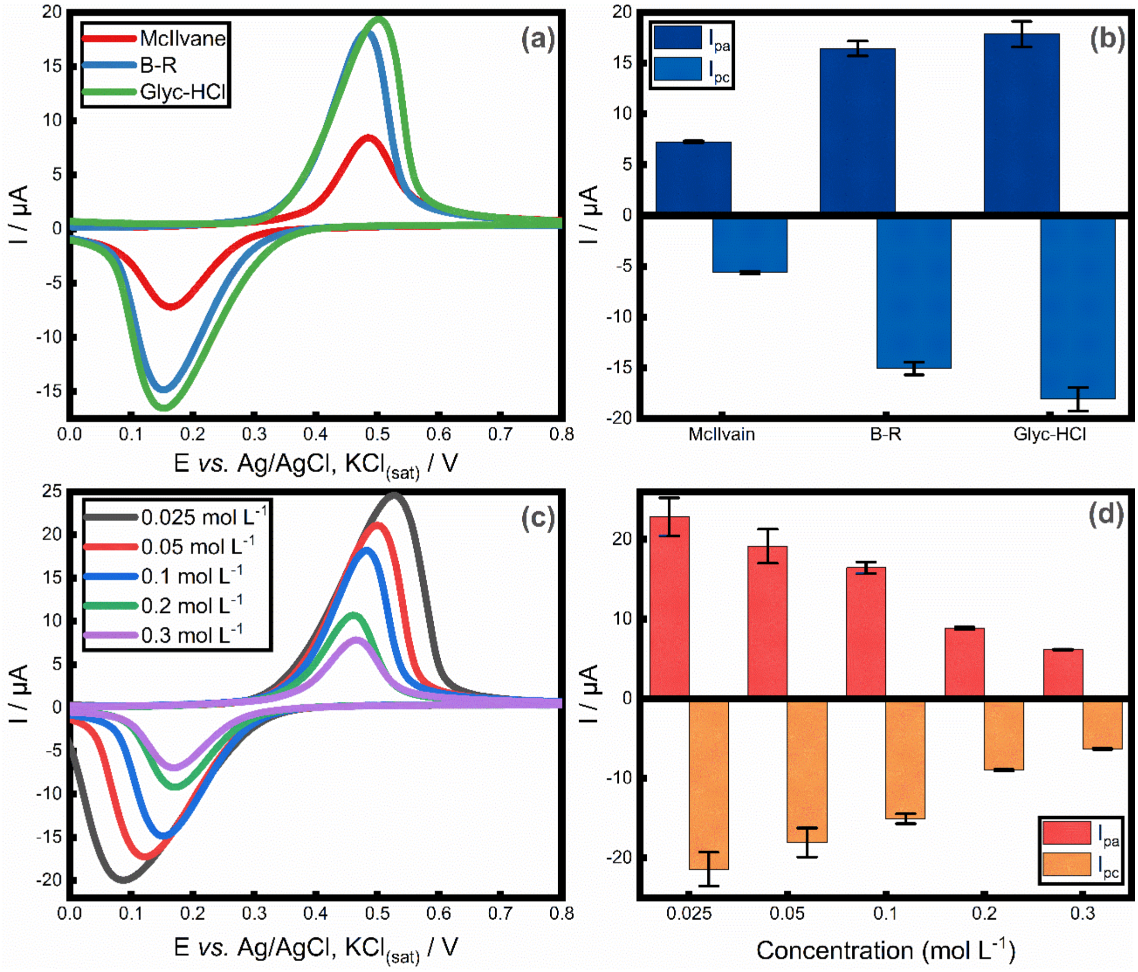

Figure S6. (a) CVs for DA 50 µmol L^{-1} on GCE/Nafion/NiNPs different buffers B-R, McIlvaine, and Glyc-HCl 0.1 mol L^{-1} (pH 3.0). (b) The anodic and cathodic current intensities for DA. (c) CVs for DA 50 µmol L^{-1} on GCE/Nafion/NiNPs at different concentrations of supporting electrolyte, and (d) the anodic and cathodic peak current intensities for DA *vs*. B-R buffer concentrations (pH 3.0).

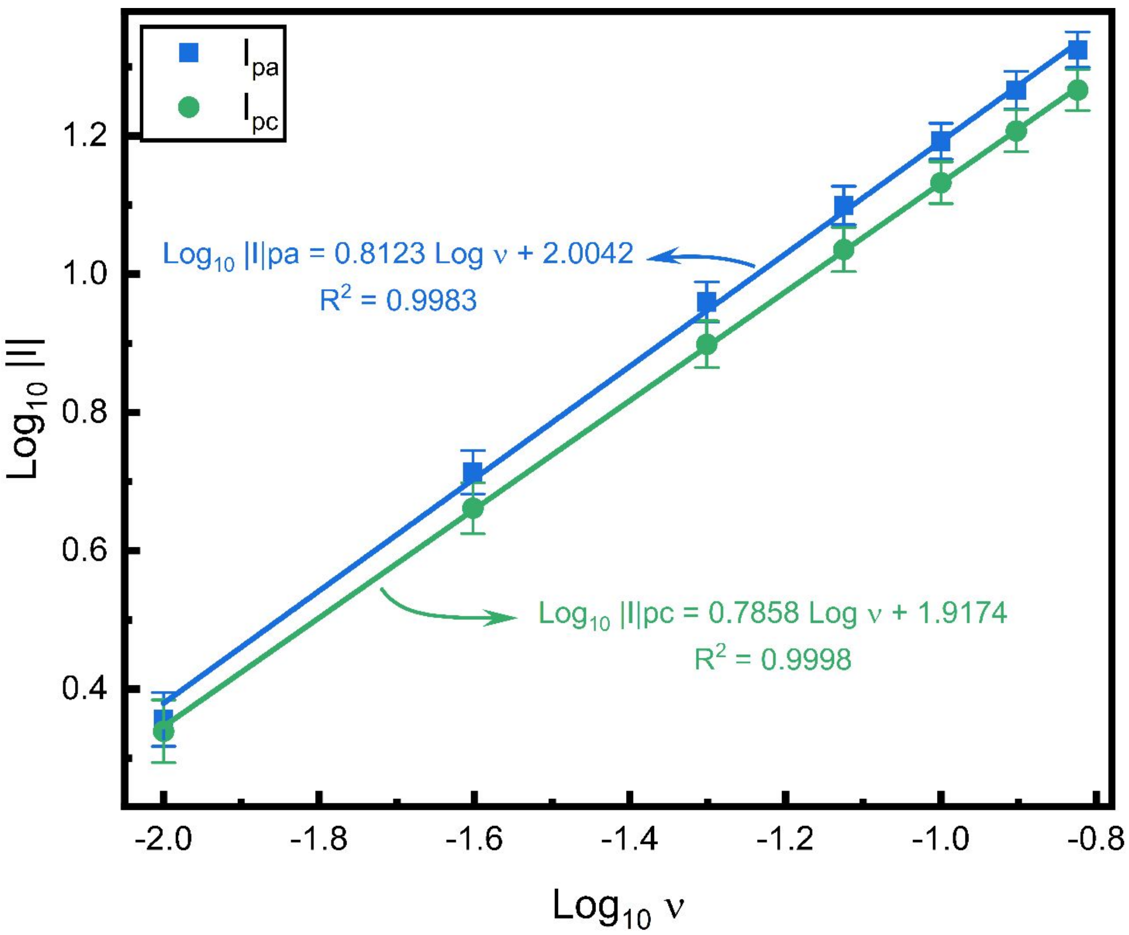

Figure S7. Effect of scanning rate studies $\log_{10}$ *v versus* $\log_{10}$ |I|.

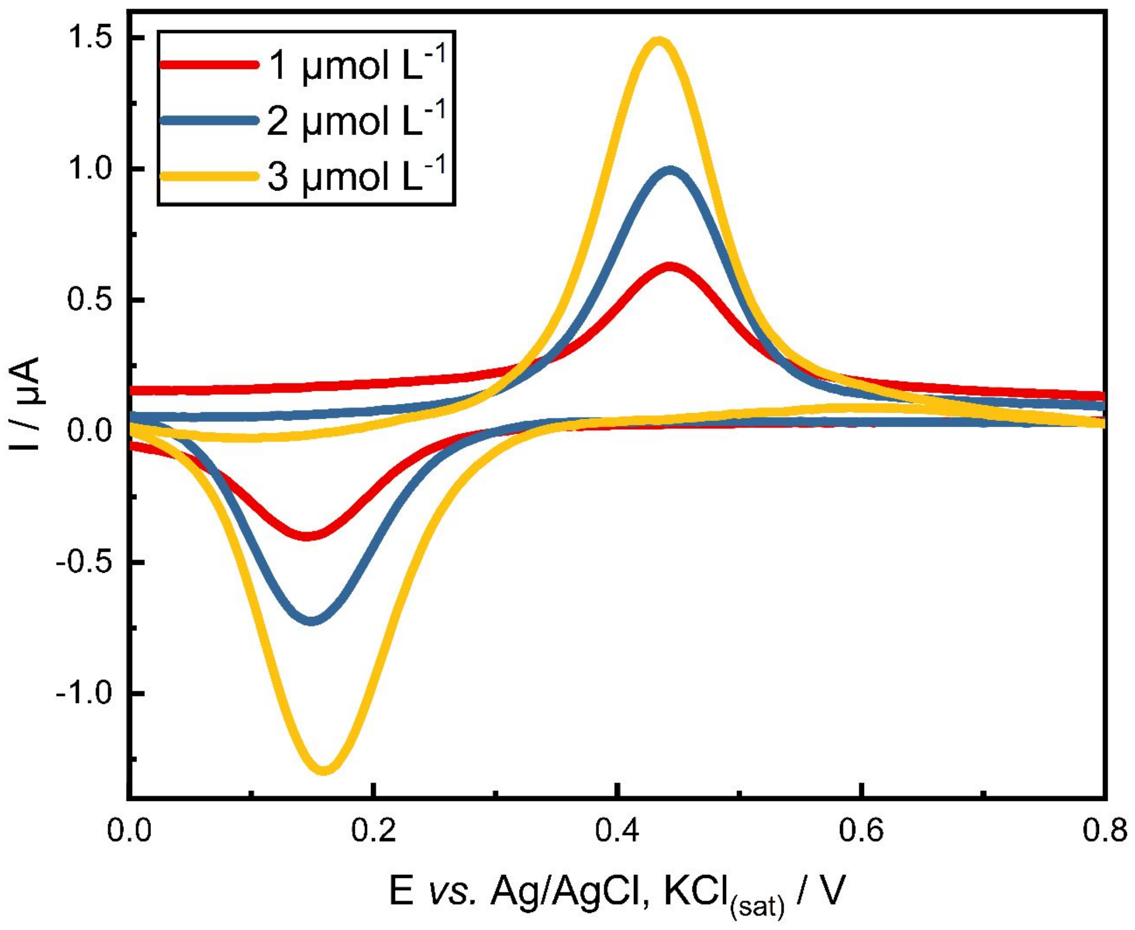

Figure S8. CV of DA in different concentration levels in the synthetic human urine medium.

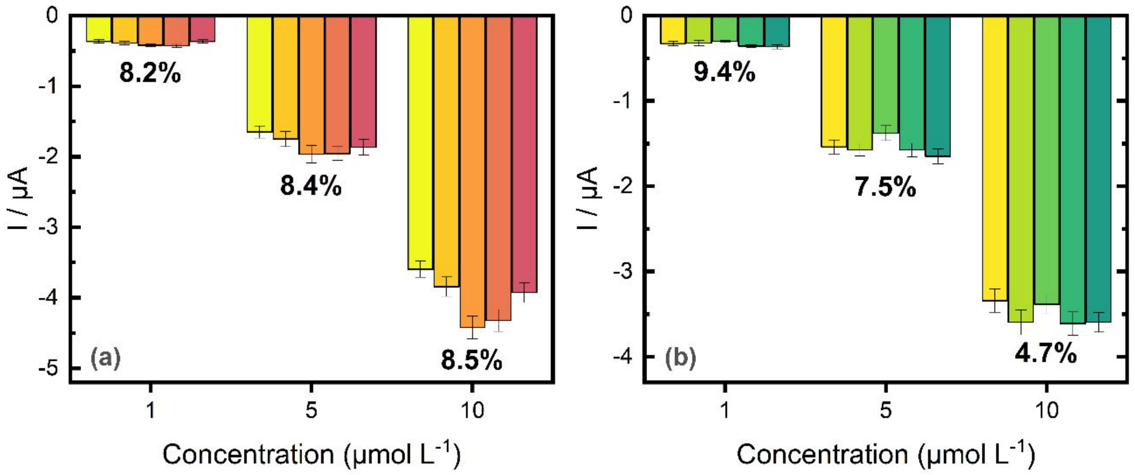

Figure S9. Repeatability studies of DA at different concentrations on GCE/Nafion/NiNPs electrochemical sensor. Data for (a) inter-day and (b) intra-day.

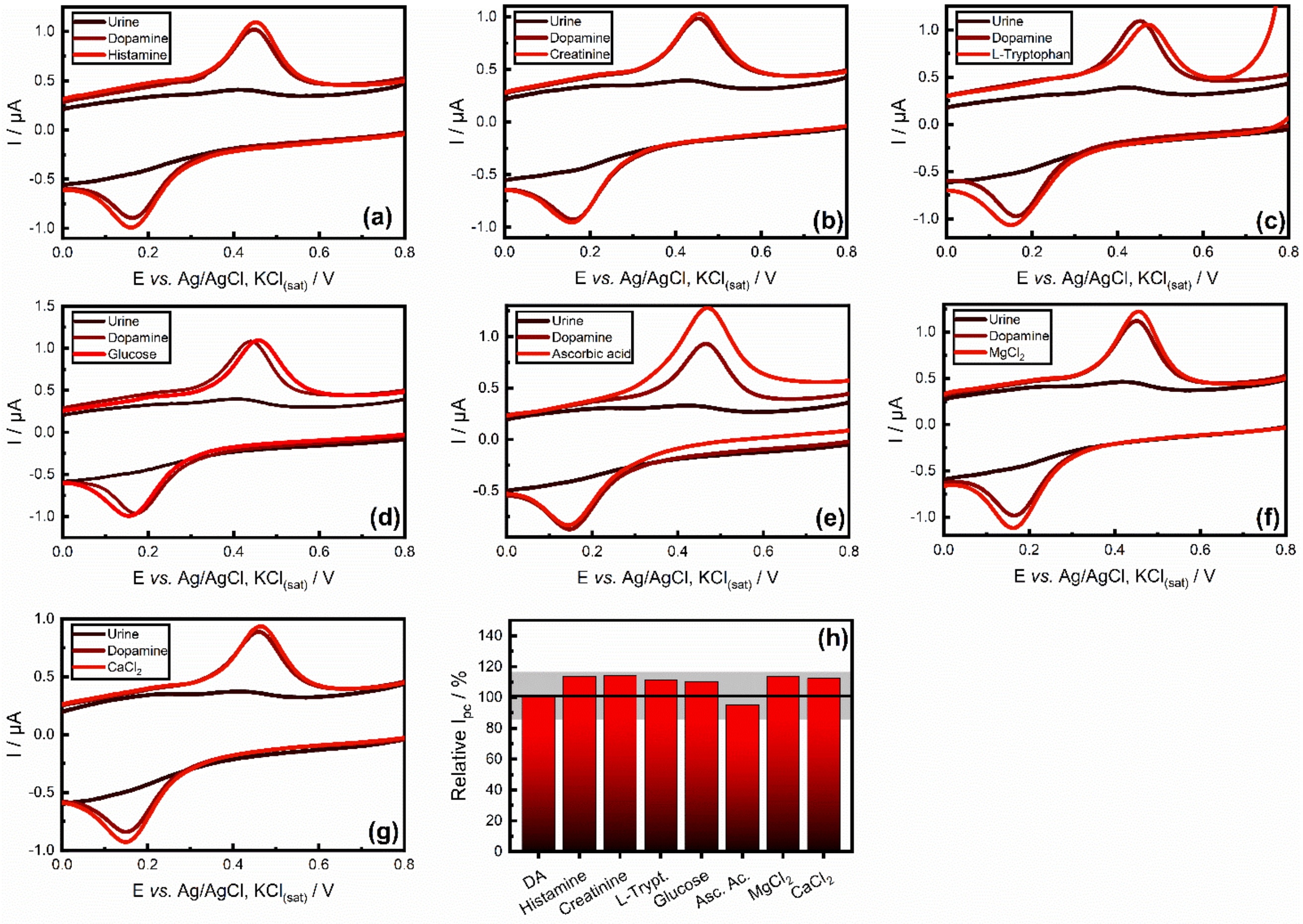

Figure S10. Selectivity studies for DA at 0.5 µmol L^{-1} on GCE/Nafion/NiNPs in synthetic human urine with additions of (a) histamine, (b) creatinine, (c) L-tryptophan, (d) glucose, (e) ascorbic acid, (f) $MgCl_2$ and (g) $CaCl_2$. (h) Relative standard cathodic peak current deviation for all substances tested.
